# PRRSV-2 nsp2 Ignites NLRP3 inflammasome through IKKβ-dependent dispersed *trans*-Golgi network translocation

**DOI:** 10.1371/journal.ppat.1012915

**Published:** 2025-01-27

**Authors:** Lujie Zhang, Yanni Gao, Haoran Zhou, Xiao Liang, Xiaolin Jiang, Wenqin Gong, Yangyang Sun, Desheng Zhang, Xianwei Wang, Hans Nauwynck, Juan Bai, Ping Jiang

**Affiliations:** 1 Key Laboratory of Animal Diseases Diagnostic and Immunology, Ministry of Agriculture, MOE International Joint Collaborative Research Laboratory for Animal Health & Food Safety, College of Veterinary Medicine, Nanjing Agricultural University, Nanjing, China; 2 Laboratory of Virology, Faculty of Veterinary Medicine, Ghent University, Salisburylaan Merelbeke, Belgium; 3 Jiangsu Co-Innovation Center for the Prevention and Control of Important Animal Infectious Disease and Zoonosis, Yangzhou University, Yangzhou, PR China; University of Michigan, USA, UNITED STATES OF AMERICA

## Abstract

The NLRP3 inflammasome is a fundamental component of the innate immune system, yet its excessive activation is intricately associated with viral pathogenesis. Porcine reproductive and respiratory syndrome virus type 2 (PRRSV-2), belonging to the family *Arteriviridae*, triggers dysregulated cytokine release and interstitial pneumonia, which can quickly escalate to acute respiratory distress and death. However, a mechanistic understanding of PRRSV-2 progression remains unclear. Here, we screen that PRRSV-2 nsp2 activates the NLRP3 inflammasome, thereby instigating a state of hyperinflammation. Mechanistically, PRRSV-2 nsp2 interacts with the nucleotide-binding and oligomerization (NACHT) domain of NLRP3, augmenting IKKβ recruitment to driving NLRP3 translocation to the dispersed *trans*-Golgi network (dTGN) for oligomerization. This process facilitates ASC polymerization, culminating in the activation of the NLRP3 inflammasome. In addition, the IKKβ-dependent NLRP3 translocation to the dTGN is pivotal for pseudorabies virus (PRV) and encephalomyocarditis virus (EMCV)-induced inflammatory responses. Collectively, these results elucidate a novel mechanism of NLRP3 inflammasome activation during PRRSV-2 infection, providing valuable insights into PRRSV-2 pathogenesis.

## Author summary

Hyperactivation of the NLRP3 inflammasome triggers excessive proinflammatory cytokine release, skewing the host response toward hyperinflammation and severe tissue damage. PRRSV-2, a positive-stranded RNA virus that causes significant economic losses in the swine industry, triggers severe inflammation and cytopathology during infection. Gaining a deep understanding of its pathogenesis is crucial for developing effective strategies to combat the disease. Here, we show that PRRSV-2 nsp2 plays a crucial role in activating the NLRP3 inflammasome by interacting with NLRP3 and facilitating the recruitment of IKKβ, which drives the translocation of NLRP3 to the dTGN for oligomerization, ultimately leading to the activation of the NLRP3 inflammasome. This process is also crucial for the inflammatory responses in both PRV and EMCV infections. Our findings uncover a novel function of PRRSV-2 nsp2 in the activation of inflammatory responses, advancing our understanding of PRRSV-2 pathogenesis, and highlight the importance of IKKβ-dependent translocation of NLRP3 to the dTGN in virus-induced NLRP3 inflammasome activation.

## Introduction

Inflammasomes are multiprotein complexes of the innate immune system, which become activated during cellular stress and infections [1,2]. Nucleotide-binding domain (NBD), leucine-rich repeat (LRR), and pyrin domain (PYD)-containing protein 3 (NLRP3) is a crucial inflammasome sensor. NLRP3 inflammasome activation is regulated through a two-step process: priming at transcriptional and posttranslational levels (signal 1), followed by assembly in response to exogenous pathogens or endogenous danger signals (signal 2) [3]. Upon activation, NLRP3 is recruited to the dTGN and undergoes oligomerization, promoting ASC (apoptosis-associated speck-like protein with a caspase recruitment domain) polymerization via its pyrin domain, which ultimately recruits Caspase-1 to activate the downstream signaling cascade [4–7]. Aberrant activation of the NLRP3 inflammasome triggers the release of pro-inflammatory cytokines, including IL-1β, IL-18, and IL-6, potentially leading to a "cytokine storm" in acute inflammatory diseases and viral infections, such as SARS-CoV-2, HSV-1, and influenza [8–10]. Consequently, the NLRP3 inflammasome functions as a "double-edged sword" in the antiviral immunity.

The IKKβ, a component of the canonical IKK complex, is a serine-threonine protein kinase that phosphorylates IκB proteins on Ser32 and Ser36, leading to their ubiquitination and degradation. As a result, NF-κB dissociates from the NF-κB/IκB complex, and translocates to nucleus, where it stimulates the transcriptions of cytokines and cell adhesion molecules [11]. Once the serine on the activation loop of IKKβ is mutated to alanine, TNF-α, IL-1, and LPS all fail to activate NF-κB [12]. In summary, the IKKβ/NF-κB pathway plays a pivotal role in pro-inflammatory responses.

Porcine reproductive and respiratory syndrome viruses (PRRSVs) are destructive swine pathogens classified as positive-stranded RNA viruses in the *Arteriviridae* family of the *Nidovirales* order, consisting of two genetically distinct species: PRRSV type 1 (PRRSV-1) (*Betaarterivirus suid 1*) and type 2 (PRRSV-2) (*Betaarterivirus suid* 2) [13–15]. PRRSV-2 infection induces severe pathology, pulmonary lesions, immune dysregulation, and elevated inflammatory cytokine levels, closely resembling the clinical features of SARS-CoV-2 and highly pathogenic avian influenza, indicating an exaggerated proinflammatory activity of PRRSV-2 [16–23]. However, whether the inflammatory lung injury induced by PRRSV-2 is mediated by excessive activation of the NLRP3 inflammasome remains enigmatic.

In this study, we uncovered a novel mechanism of NLRP3 activation during PRRSV-2 infection, in which nsp2 interacts with the NACHT domain of NLRP3, intensifying IKKβ recruitment, directing NLRP3 translocation to the dTGN, and enabling ASC polymerization to trigger inflammasome activation. These findings provide critical insights for PRRSV-2 pathogenesis and highlight the critical role of IKKβ in virus-induced inflammation.

## Results

### PRRSV-2 triggers inflammatory responses by activating NLRP3 inflammasome

**Fig 1:**
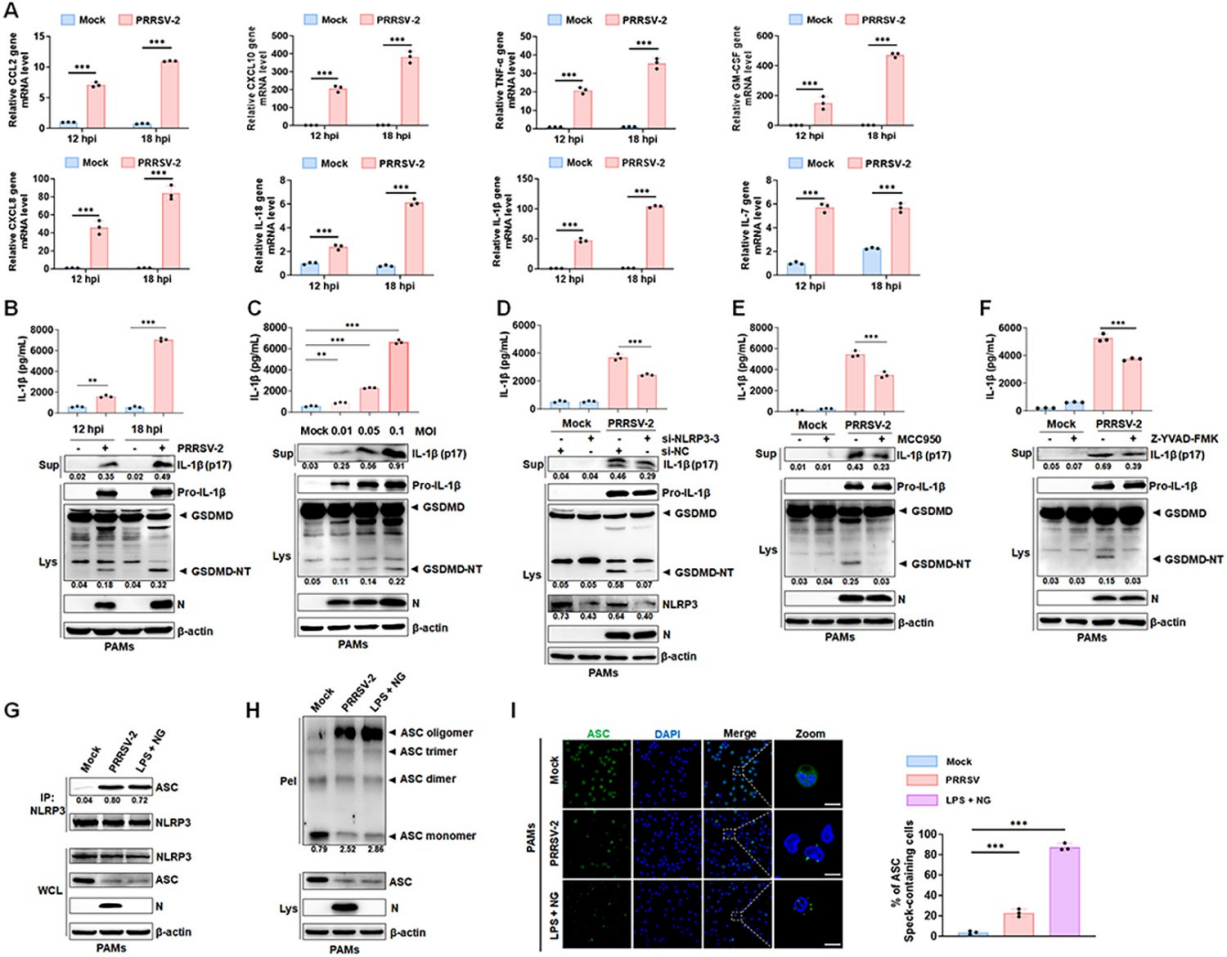
PRRSV-2 triggers inflammatory responses by activating NLRP3 inflammasome. (A) PAMs infected with PRRSV-2 at an MOI of 0.05 for 12 h or 18 h, and gene transcription levels were evaluated by qRT-PCR. The relative expression changes were calculated by using 2^^(-ΔΔCt)^ method. (B and C) The mature IL-1β (p17) levels in PAMs supernatants were assessed by ELISA and Western blot after PRRSV-2 infection at MOI of 0.05 for 12 or 18 hours (B), and at MOI of 0.01, 0.05, or 0.1 for 18 hours (C). The pro-IL-1β and GSDMD in cell lysates were analyzed by Western blot. (D) PAMs transfected with control siRNA or siRNA targeting NLRP3 (si-NLRP3-3) were infected with PRRSV-2 (MOI of 0.05) for 18 hours. The mature IL-1β (p17) levels in supernatants were measured by ELISA and Western blot, and pro-IL-1β and GSDMD in cell lysates were detected by Western blot. (E and F) PAMs were pre-treated with 50 μM MCC950 (E) or Z-YVAD-FMK (F) for 1 hour, then infected with PRRSV-2 at an MOI of 0.05 for 18 hours. The pro-IL-1β and GSDMD in lysates were analyzed by Western blot, and the mature IL-1β (p17) levels in supernatants were measured by ELISA and Western blot. (G) PAMs infected with PRRSV-2 (MOI of 0.05) for 18 hours or treated with LPS and Nigericin were analyzed by immunoprecipitation using an anti-NLRP3 antibody. (H and I) PAMs were infected with PRRSV-2 at an MOI of 0.05 for 18 h or treated with 100 ng/mL LPS for 8 h followed by 10 μM Nigericin for another 4 h as a positive control. ASC oligomerization was analyzed by cross-linking the cytosolic pellets and visualized by Western blot (H). ASC speck formation in mock, PRRSV-2-infected, and LPS/Nigericin-treated PAMs was observed by confocal microscopy (I, left). Percentages of ASC speck-positive cells are shown (I, right). NG, Nigericin; Sup, supernatant; WCL: whole cell lysate; Lys, lysate; Pel, pellets. For confocal microscopy assay, results were manually quantified from at least 30 randomly selected cells per condition per replicate using Image J. Scale bar: 10 μm. The protein levels were quantified by Image J and normalized to β-actin. The data are representative of results from three independent experiments. Error bars indicate the mean (± SD) of three repeats. *p ≤ 0.05, **p ≤ 0.01, ***p ≤ 0.001; p > 0.05 stands for ns.

Interstitial pneumonia is frequently observed in PRRSV-2-infected pigs, and inflammation plays a crucial role in the development of PRRSV-2-related pathology [17,20]. To assess whether PRRSV-2 infection induces an inflammatory response in porcine alveolar macrophages (PAMs), mRNA expression levels of various pro-inflammatory cytokines were analyzed. At each time point, PRRSV-2-infected PAMs showed significantly elevated expression of proinflammatory cytokines (IL-18, IL-1β and TNF-α) and chemokines (C–C motif chemokine ligand 2 (CCL2), C-X-C motif chemokine ligand 8 (CXCL8), CXCL10, GM-CSF, and IL-7) compared to the control groups (Fig 1A). The Gasdermin D (GSDMD) cleavage and IL-1β production indicated that PRRSV-2 induced pyroptosis and facilitated the maturation and secretion of IL-1β in a dose- and time-dependent manner (Fig 1B and 1C). To examine the role of NLRP3 in PRRSV-2-induced inflammatory responses, we used RNA interference and MCC950 [24], a specific inhibitor of NLRP3, to suppress its expression and activity, respectively, resulting in a reduction in mature IL-1β secretion and attenuated GSDMD cleavage (Fig 1D and 1E). And Z-YVAD-FMK, a Caspase-1 inhibitor, also significantly suppressed PRRSV-2-induced IL-1β maturation and release, along with pyroptosis in PAMs (Fig 1F). As a key downstream component in NLRP3 inflammasome activation, ASC was investigated for its role in PRRSV-2-induced inflammatory responses [25]. As shown in Fig 1G–1H, PRRSV-2 infection enhanced the interaction between NLRP3 and ASC, promoting NLRP3 inflammasome assembly in PAMs, similar to the effect of LPS plus nigericin (Fig 1G). Western blot analysis revealed ASC oligomerization in PRRSV-2-infected or LPS plus nigericin-treated PAMs (Fig 1H). Meanwhile, ASC formed distinct specks in the cytoplasm of these cells, contrasting with its diffuse distribution in mock cells (Fig 1I). To confirm whether PRRSV-2 induces the inflammatory responses via the NLRP3 inflammasome, we inhibited NLRP3 with MCC950, which significantly reduced CXCL8, CXCL10, GM-CSF, IL-18, and IL-1β mRNA levels, but not CCL2, IL-7, and TNF-α mRNA levels (S2 Fig) during PRRSV-2 infection. Collectively, these findings suggested that PRRSV-2 infection activated NLRP3 inflammasome, contributing to the cytokine storm and pyroptosis.

### PRRSV-2 nsp2 promotes the activation of NLRP3 inflammasome

**Fig 2:**
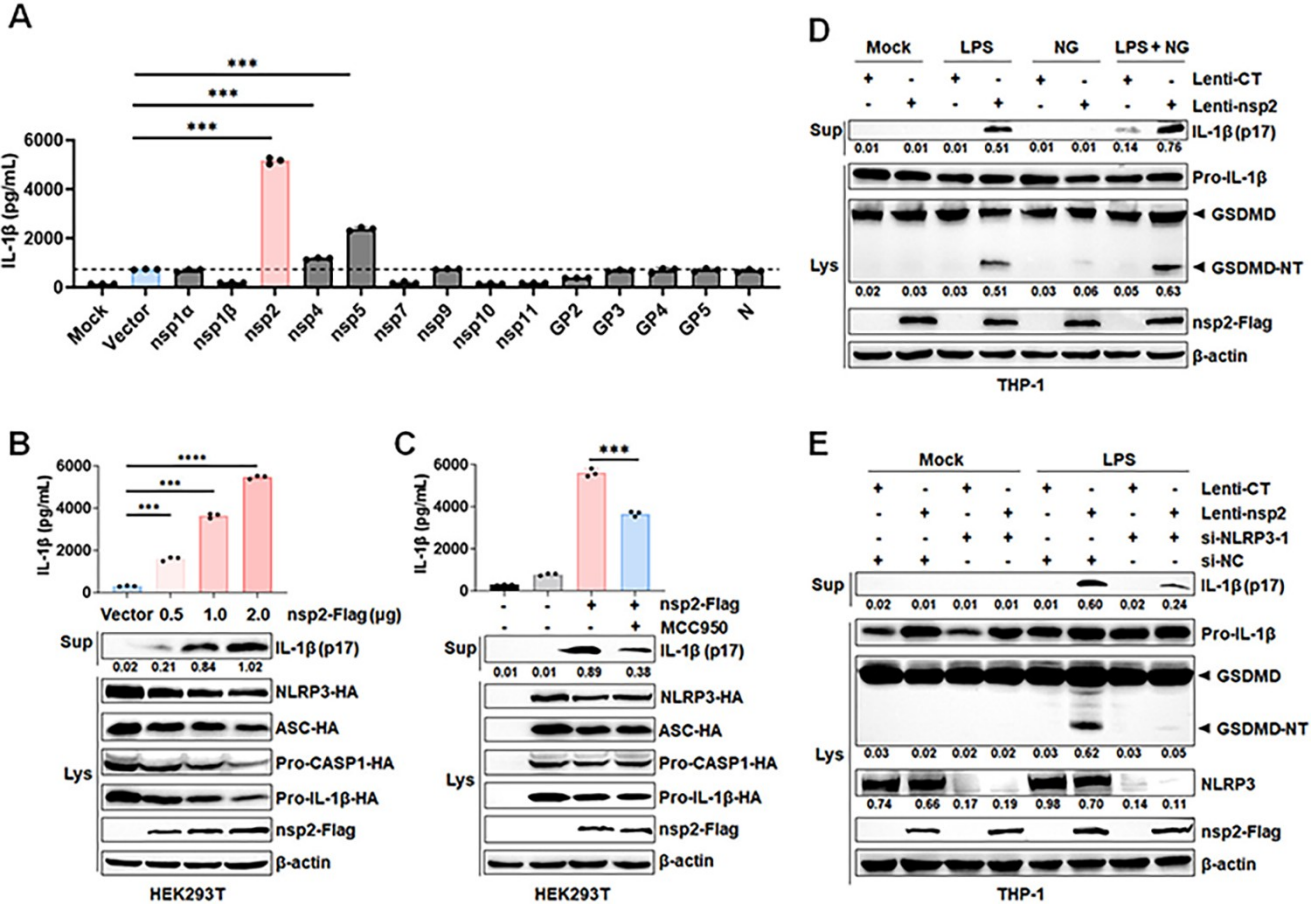
PRRSV-2 nsp2 promotes the activation of NLRP3 inflammasome. (A) HEK293T cells co-transfected with HA-tagged NLRP3, ASC, pro-CASP1, pro-IL-1β, and Flag-tagged PRRSV-2 protein plasmids or empty vector (pCAGGS) for 24 h were analyzed for IL-1β in supernatants by ELISA. (B) HEK293T cells co-transfected with HA-tagged NLRP3, ASC, pro-CASP1, pro-IL-1β, and varying concentrations of Flag-tagged nsp2 plasmids or pCAGGS for 24 h were analyzed for IL-1β in supernatants by ELISA and mature IL-1β (p17) in supernatants by Western blot. (C) HEK293T cells co-transfected with HA-tagged NLRP3, ASC, pro-CASP1, pro-IL-1β, and Flag-tagged nsp2 or pCAGGS. After 6 h, HEK293T cells were treated with 50 μM MCC950 for 18 h. Supernatants were analyzed for IL-1β by ELISA, and mature IL-1β (p17) by Western blot. (D) THP-1 cells infected with Lentivirus-CT or Lentivirus-nsp2, differentiated with PMA, then treated with either 1 μg/mL LPS for 12 h, 2 μM Nigericin for 1 h, or LPS (12 h) followed by Nigericin (1 h). Mature IL-1β (p17) in supernatants and GSDMD in lysates were analyzed by Western blot. (E) THP-1 cells were stably infected with Lentivirus-CT or Lentivirus-nsp2, differentiated with PMA. Then, THP-1 were transfected with control siRNA (si-NC) or siRNA targeting NLRP3 (si-NLRP3-1) 100 nM for 24 h, followed by stimulation with 1 μg/mL LPS for 12 h. Mature IL-1β (p17) in supernatants and GSDMD in lysates were determined by Western blot. Lenti, Lentivirus; CT, Control; NG, Nigericin; Sup, supernatant; Lys, lysate. The protein levels were quantified by Image J and normalized to β-actin. The data are representative of results from three independent experiments. Error bars indicate the mean (± SD) of three repeats. *p ≤ 0.05, **p ≤ 0.01, ***p ≤ 0.001; p > 0.05 stands for ns.

The PRRSVs genome, consisting of 11 known open reading frames (ORFs), encodes 13 nonstructural proteins (nsps), including nsp1α, nsp1β, and nsp2 to nsp12, as well as 6 structural proteins [26]. To screen the specific viral proteins responsible for NLRP3 inflammasome activation, HEK293T cells were used to construct an NLRP3 inflammasome system by co-transfection with the plasmids encoding NLRP3, ASC, and pro-Caspase-1 of the NLRP3 inflammasome, along with the substrate pro-IL-1β. Next, each PRRSV-2 non-structural and structural protein recombinant plasmid were transfected into the HEK293T-NLRP3 inflammasome system cells. ELISA results revealed that nsp2, nsp4, and nsp5 induced IL-1β secretion, with nsp2 exhibiting the highest activity (Figs 2A and S3A). Further analysis revealed that nsp2 promotes IL-1β maturation and secretion in a dose-dependent manner, which can be inhibited by MCC950 (Fig 2B and 2C).

NLRP3 inflammasome activation requires two steps, both a priming step (e.g., LPS, signal 1) and an activation step (e.g., nigericin, signal 2) [3]. To determine the stage at which nsp2 acts, THP-1 cells were infected with Lentivirus-nsp2, differentiated into macrophages, and treated with lipopolysaccharide (LPS), nigericin (NG), or both. Nsp2 alone fails to induce pyroptosis or IL-1β maturation and secretion. However, in the presence of LPS, it promotes both processes and further amplifies them when exposed to LPS plus NG, indicating nsp2 primarily functions at the NLRP3 inflammasome activation stage (Fig 2D). Furthermore, to elucidate the role of NLRP3 involved in nsp2-induced mature IL-1β secretion and pyroptosis, we suppressed its expression via RNA interference and inhibited its activity with MCC950, resulting in a marked reduction in IL-1β production and GSDMD cleavage in THP-1 cells (Figs 2E, S1B and S3B). Collectively, these results suggested that PRRSV-2 nsp2 plays a pivotal role in NLRP3 inflammasome activation.

### PRRSV-2 nsp2 TM domain interacts with NLRP3 to promote IL-1β maturation and secretion

**Fig 3:**
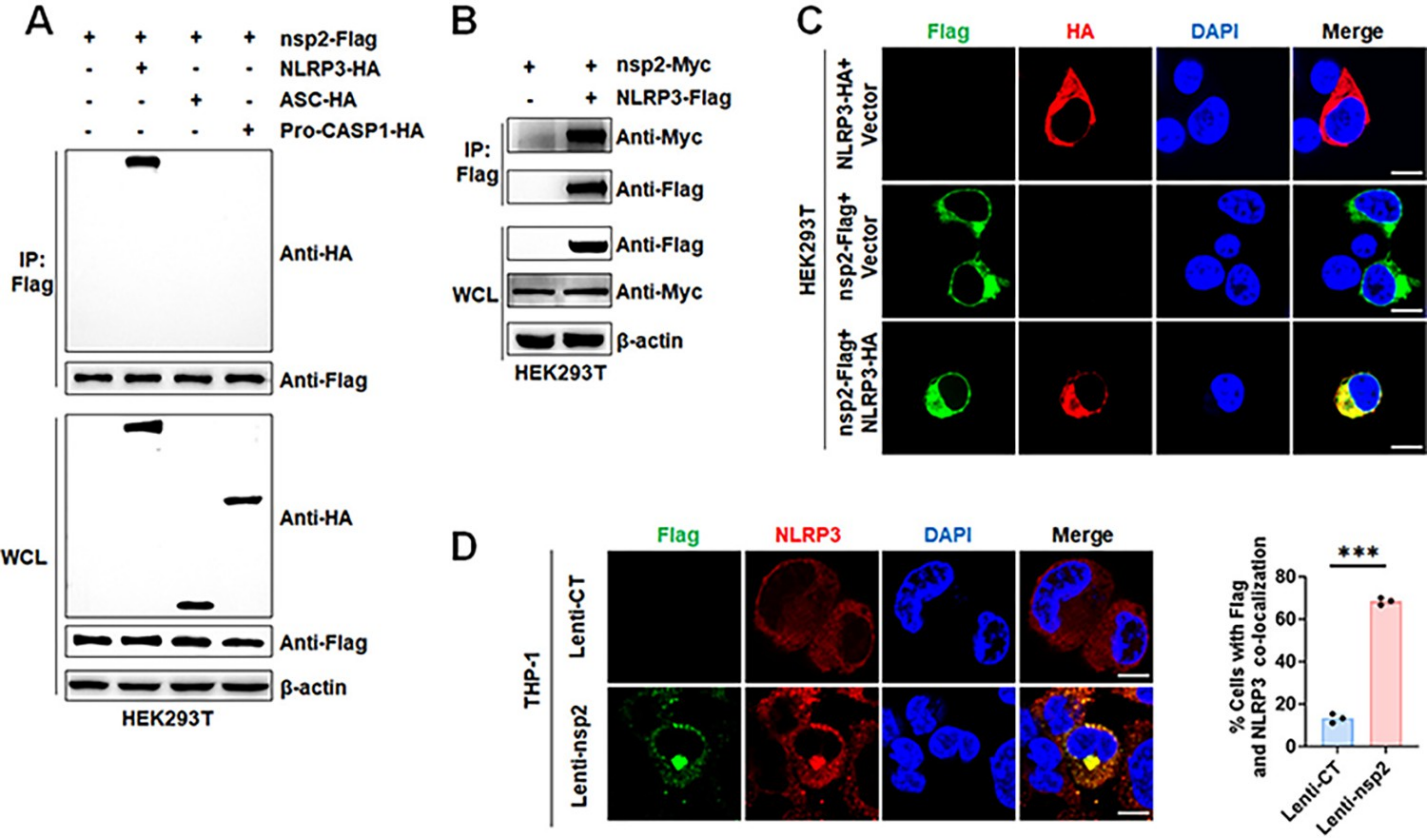
PRRSV-2 nsp2 interacts with NLRP3 protein. (A) HEK293T cells co-transfected with Flag-tagged nsp2, empty vector (pCAGGS), HA-tagged NLRP3, ASC, or pro-CASP1 for 24 h were immunoprecipitated with an anti-Flag antibody. (B) HEK293T cells co-transfected with Flag-tagged NLRP3 and either empty vector (pCAGGS) or Myc-tagged nsp2 for 24 h were immunoprecipitated with an anti-Flag antibody. (C) HEK293T cells transfected with Flag-tagged nsp2 or empty vector (pCAGGS) and HA-tagged NLRP3 were visualized with confocal microscopy for DAPI (blue), nsp2 (green), and NLRP3 (red). (D) THP-1 cells were stably infected with Lentivirus-CT or Lntivirus-nsp2, differentiated with PMA, were stained for Flag-tagged nsp2 and NLRP3. Representative immunofluorescence images showing nsp2-Flag and NLRP3 co-localization were presented on the (D, left). The percentages of nsp2-Flag and NLRP3 co-localization containing cells were presented on the (D, right). WCL, whole cell lysate. For confocal microscopy assay, results were manually quantified from at least 30 randomly selected cells per condition per replicate using Image J. Scale bar: 10 μm. The data are representative of results from three independent experiments. Error bars indicate the mean (± SD) of three repeats. *p ≤ 0.05, **p ≤ 0.01, ***p ≤ 0.001; p > 0.05 stands for ns.

To explore the mechanism by which nsp2 activates NLRP3 inflammasome, the interactions between nsp2 and NLRP3, ASC, or pro-Caspase-1 were detected. Co-IP assays demonstrated that nsp2 specifically interacted with NLRP3 but not with ASC or pro-Caspase-1 (Fig 3A), which was also confirmed by reciprocal Co-IP assays (Fig 3B). Confocal microscopy analysis demonstrated the cytoplasmic co-localization between nsp2 and NLRP3 both exogenously and endogenously in nsp2/NLRP3-overexpressed HEK293T cells (Fig 3C) and Lentivirus-nsp2-infected THP-1 cells (Fig 3D). These results suggested that nsp2 selectively interacts with NLRP3, thereby modulating NLRP3 inflammasome formation.

**Fig 4:**
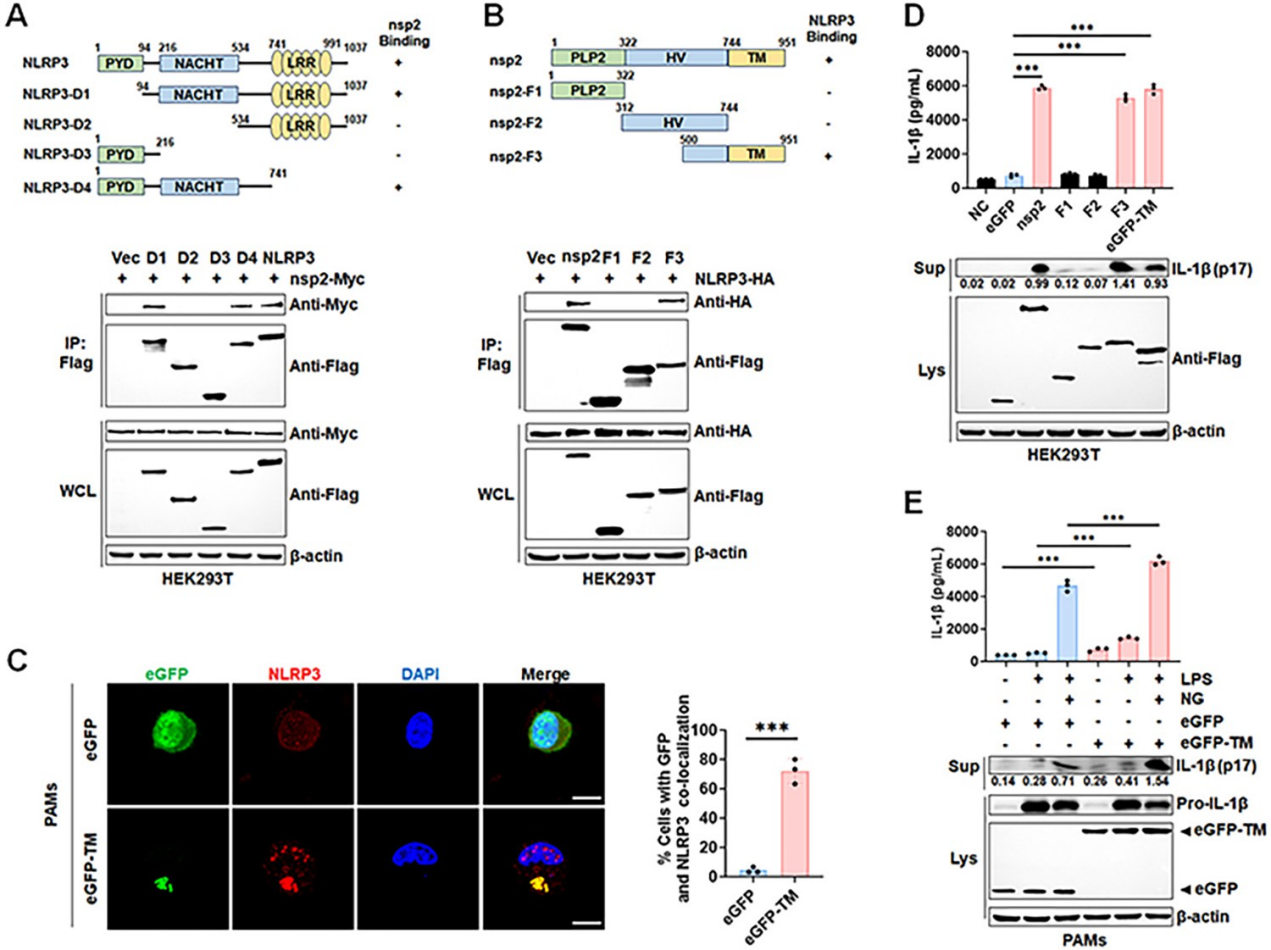
PRRSV-2 nsp2 TM domain is crucial for NLRP3 inflammasome activation. (A) HEK293T cells were co-transfected with a plasmid encoding Myc-tagged nsp2, either alone or with a plasmid encoding Flag-tagged NLRP3 or its truncated mutants for 24 h. Cell lysates were immunoprecipitated using an anti-Flag antibody. (B) HEK293T cells were co-transfected with a plasmid encoding HA-tagged NLRP3, either alone or with a plasmid encoding Flag-tagged nsp2 or its truncated mutants for 24 h. Cell lysates were immunoprecipitated using an anti-Flag antibody. (C) PAMs were transfected with eGFP or eGFP-TM mRNA for 24 h. Confocal microscopy images show eGFP-TM and NLRP3 co-localization (C, left), with quantification of co-localized cells on (C, right). (D) HEK293T cells were co-transfected with plasmids encoding HA-tagged NLRP3, ASC, pro-CASP1, pro-IL-1β, and Flag-tagged nsp2 or its truncated mutants for 24 h. IL-1β levels in supernatants were measured by ELISA, and mature IL-1β (p17) in supernatants and protein expression were analyzed by Western blot. (E) PAMs were transfected with eGFP or eGFP-TM mRNA for 24 h, followed by treatment with 100 ng/mL LPS for 8 h and 10 μM Nigericin for 4 h. IL-1β levels in supernatants were measured by ELISA, and mature IL-1β (p17) in supernatants and protein expression were analyzed by Western blot. WCL, whole cell lysate; NG, Nigericin; Sup, supernatant; Lys, lysate. For confocal microscopy assay, results were manually quantified from at least 30 randomly selected cells per condition per replicate using Image J. Scale bar: 10 μm. The protein levels were quantified by Image J and normalized to β-actin. The data are representative of results from three independent experiments. Error bars indicate the mean (± SD) of three repeats. *p ≤ 0.05, **p ≤ 0.01, ***p ≤ 0.001; p > 0.05 stands for ns.

Nsp2 is the largest viral protein (117–131 kDa), with three domains: an N-terminal cysteine protease (PLP2), a hypervariable region (HV), and a C-terminal transmembrane domain (TM), followed by a C-terminal tail of uncertain size [27]. To delineate the domain essential for NLRP3 interaction with nsp2, Co-IP results indicated that the NACHT domain of NLRP3 and TM domain of nsp2 were crucial for NLRP3-nsp2 interaction (Fig 4A and 4B). Confocal microscopy analysis confirmed the cytoplasmic co-localization between nsp2 TM domain and NLRP3 in eGFP-TM mRNA transfected PAMs (Fig 4C). The impact of nsp2 truncations on NLRP3 inflammasome activation were also assessed. HEK293T-NLRP3 inflammasome system cells were transfected with the plasmids encoding nsp2 or its truncated mutants. The results revealed that IL-1β secretion and IL-1β p17 cleavage in the cell supernatants were significantly induced by nsp2 and its TM domain, but not PLP2 and the HV domain (Fig 4D). Notably, the nsp2 TM domain enhanced LPS or LPS plus NG-induced maturation and secretion of IL-1β in PAMs (Fig 4E). These results suggested that the TM domain of nsp2 is essential for NLRP3 inflammasome activation.

### PRRSV-2 nsp2 to facilitates NLRP3 inflammasome assembly

**Fig 5:**
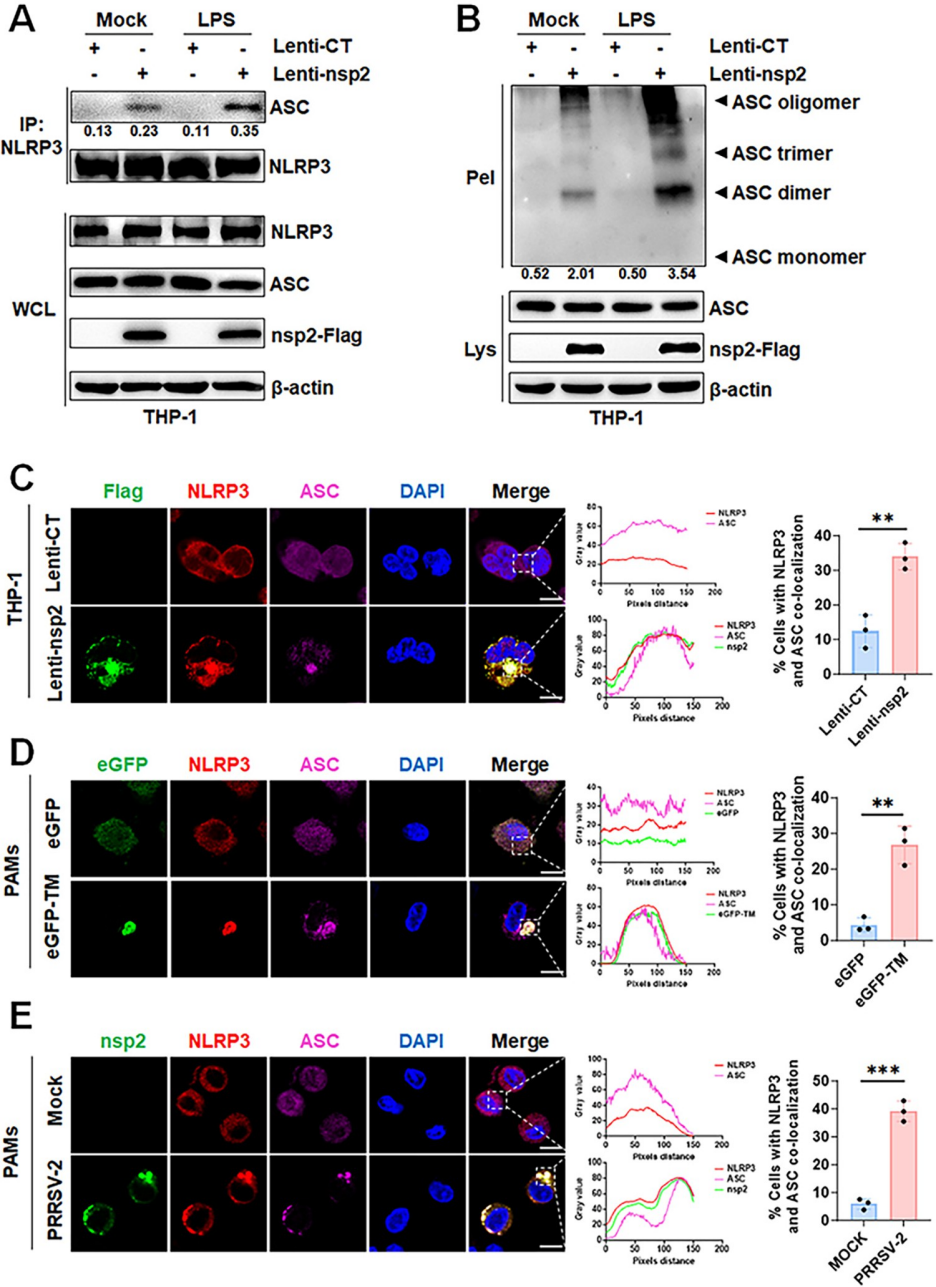
PRRSV-2 nsp2 facilitates NLRP3 inflammasome assembly. (A and B) THP-1 cells stably infected with Lentivirus-CT or Lentivirus-nsp2, differentiated with PMA, followed by stimulation with 1 μg/mL LPS or culture media for 12 h. Cell lysates were immunoprecipitated with an anti-NLRP3 antibody (A). ASC oligomerization was assessed by cross-linking cytosolic pellets and analyzed by Western blot (B). (C) THP-1 cells stably infected with Lentivirus-CT or Lentivirus-nsp2, differentiated with PMA, then stained with anti-Flag, anti-NLRP3, and anti-ASC antibodies. Confocal microscopy images showing co-localization of nsp2-Flag, NLRP3, and ASC were presented (C, left). Gray values for each channel were measured by ImageJ (C, middle), and percentages of co-localizing cells were shown (C, right). (D) PAMs were transfected with eGFP or eGFP-TM mRNA for 24 h. Confocal images showing eGFP-TM, NLRP3, and ASC co-localization were presented (D, left). Gray values were measured by ImageJ (D, middle), and percentages of co-localizing cells were shown (D, right). (E) PAMs were infected with PRRSV-2 at MOI of 0.05 for 18 h. Confocal images showing co-localization of nsp2, NLRP3, and ASC were shown (E, left). Gray values were measured by ImageJ (E, middle), and percentages of co-localizing cells were displayed (E, right). WCL, whole cell lysate; Lys, lysate; Pel, pellets. For confocal microscopy assay, results were manually quantified from at least 30 randomly selected cells per condition per replicate using Image J. Scale bar: 10 μm. The protein levels were quantified by Image J and normalized to β-actin. The data are representative of results from three independent experiments. Error bars indicate the mean (± SD) of three repeats. *p ≤ 0.05, **p ≤ 0.01, ***p ≤ 0.001; p > 0.05 stands for ns.

In the process of inflammasome assembly, activation of NLRP3 leads to the recruitment of ASC and facilitates its aggregation into oligomers [3]. To figure out the role of nsp2 on NLRP3 recruitment of ASC, THP-1 cells were infected with Lentivirus-nsp2, differentiated into macrophages, the recruitment of endogenous ASC to NLRP3 was observed following LPS stimulation. The results showed that compared to the control group, nsp2 alone induces a modest enhancement of NLRP3-ASC interaction and ASC oligomerization; however, upon LPS stimulation, nsp2 markedly intensifies these processes (Fig 5A and 5B). Moreover, confocal microscopy revealed evident co-localization of nsp2, NLRP3, and ASC, forming spherical structures in Lentivirus-nsp2-infected THP-1 cells (Fig 5C), eGFP-TM-overexpressed PAMs (Fig 5D), and PRRSV-2-infected PAMs (Fig 5E). Collectively, our results revealed that PRRSV-2 nsp2 promotes the assembly of the NLRP3 inflammasome.

### IKKβ is essential for nsp2-driven NLRP3 inflammasome activation

**Fig 6:**
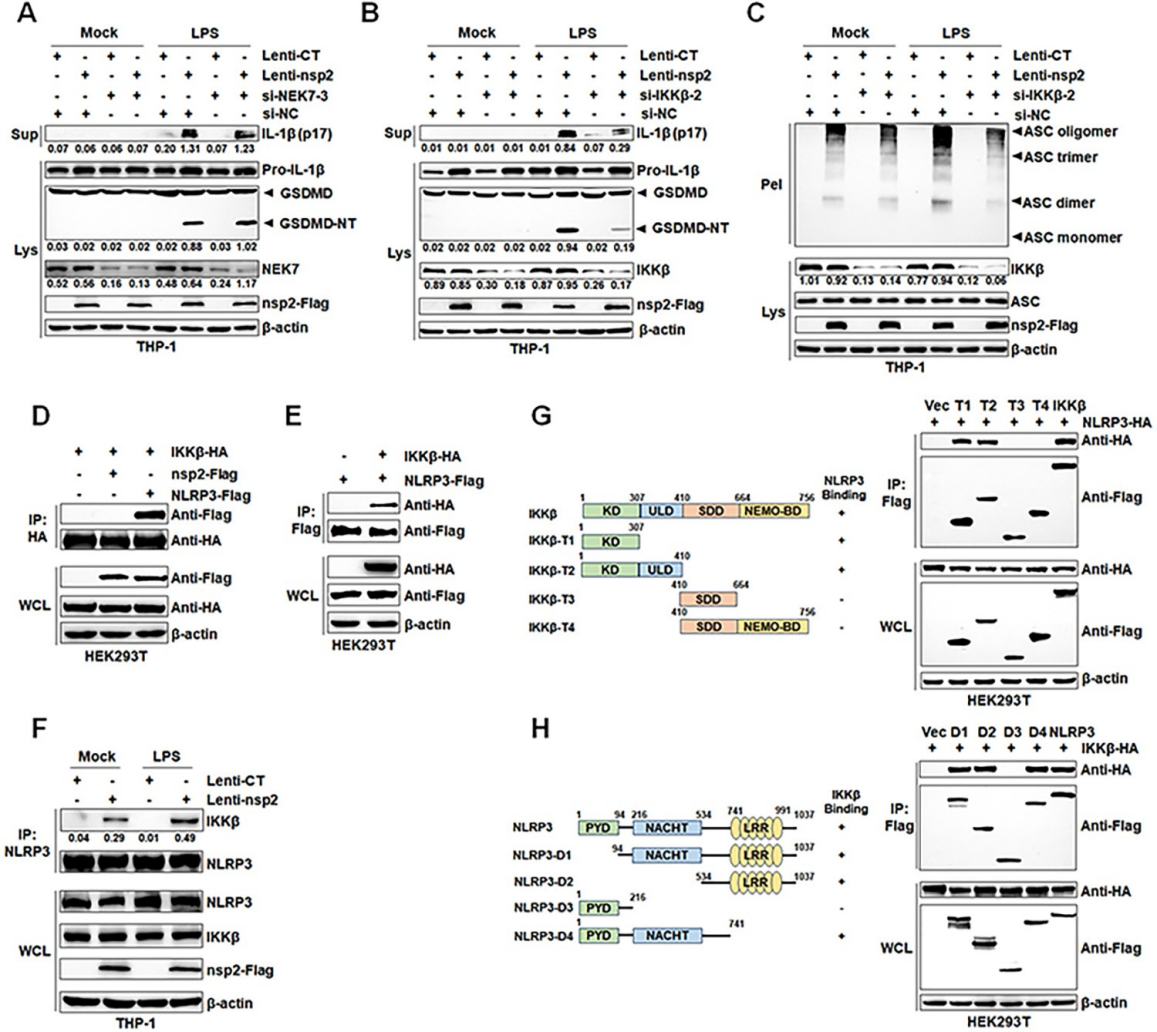
IKKβ is essential for nsp2-driven NLRP3 inflammasome activation. (A) THP-1 cells stably infected with Lentivirus-CT or Lentivirus-nsp2, differentiated with PMA, then transfected with control siRNA (si-NC) or NEK7-targeting siRNA (si-NEK7-3) at 100 nM for 24 h, followed by stimulation with 1 μg/mL LPS or culture medium for 12 h. Mature IL-1β (p17) in supernatants and GSDMD in lysates were assessed by Western blot. (B and C) THP-1 cells stably infected with Lentivirus-CT or Lentivirus-nsp2, differentiated with PMA, then transfected with control siRNA (si-NC) or IKKβ-targeting siRNA (si-IKKβ-2) at 100 nM for 24 h, followed by stimulation with 1 μg/mL LPS or culture medium for 12 h. Mature IL-1β (p17) in supernatants and GSDMD in lysates were assessed by Western blot (B). ASC oligomerization was assessed by cross-linking cytosolic pellets and visualized by Western blot (C). (D) HEK293T cells were co-transfected with HA-tagged IKKβ and either empty vector (pCAGGS), Flag-tagged nsp2 or NLRP3. Cell lysates were immunoprecipitated with an anti-HA antibody. (E) HEK293T cells were co-transfected with Flag-tagged NLRP3 and either empty vector (pCAGGS) or HA-tagged IKKβ. Cell lysates were immunoprecipitated with an anti-Flag antibody. (F) THP-1 cells stably infected with Lentivirus-CT or Lentivirus-nsp2, differentiated with PMA, followed by stimulation with 1 μg/mL LPS or culture medium for 12 h. Cell lysates were immunoprecipitated with an anti-NLRP3 antibody. (G) HEK293T cells were transfected with IKKβ-HA plasmid alone or with NLRP3-Flag or its truncated mutants. At 24 hpt, cell lysates were immunoprecipitated using an anti-Flag antibody. (H) HEK293T cells were transfected with NLRP3-HA plasmid alone or with IKKβ-Flag or its truncated mutants. At 24 hpt, cell lysates were immunoprecipitated using an anti-Flag antibody. WCL, whole cell lysate; Sup, supernatant; Lys, lysate; Pel, pellets. The protein levels were quantified by Image J and normalized to β-actin. The data are representative of results from three independent experiments.

Previous studies have demonstrated that NEK7 is crucial for facilitating NLRP3 inflammasome activation [28], the role of NEK7 on nsp2-mediated NLRP3 inflammasome activation was explored. The results showed that the siRNA targeting NEK7 was unable to inhibit nsp2-induced pyroptosis and IL-1β maturation and secretion in PMA-differentiated THP-1 cells (Figs 6A and S1C). Interestingly, recent studies have found that transcription-independent NLRP3 activation occurs by circumventing NEK7 via IKKβ [29–31]. To determine the role of IKKβ in nsp2-driven NLRP3 inflammasome activation, we suppressed its expression via RNA interference and inhibited its activity with TPCA-1, resulting in a marked reduction in nsp2-induced GSDMD cleavage, mature IL-1β secretion, and ASC oligomerization in PMA-differentiated THP-1 macrophages (Figs 6B, 6C, S1D and S4A).

To elucidate the mechanism through which nsp2 employs IKKβ to instigate NLRP3 inflammasome activation, the interactions between IKKβ and nsp2 or NLRP3 were tested. Co-IP results demonstrated that IKKβ specifically interacted with NLRP3 but not nsp2, which was confirmed by the reciprocal Co-IP assays (Fig 6D and 6E). Notably, endogenous IP result showed that, compared to the control group, nsp2 enhanced the interaction between NLRP3 and IKKβ in PMA-differentiated THP-1 macrophages, and LPS stimulation further promoted this interaction (Fig 6F). Additionally, the critical domains responsible for this interaction were identified as the NACHT and LRR domains of NLRP3 and the N-terminal kinase domain (KD) of IKKβ (Fig 6G and 6H). Taken together, these results indicated that nsp2 activates the NLRP3 inflammasome by enhancing NLRP3 interaction with IKKβ, rendering NEK7 unnecessary for this activation process.

### PRRSV-2 nsp2 promotes NLRP3 translocation to dTGN and oligomerization through IKKβ

**Fig 7:**
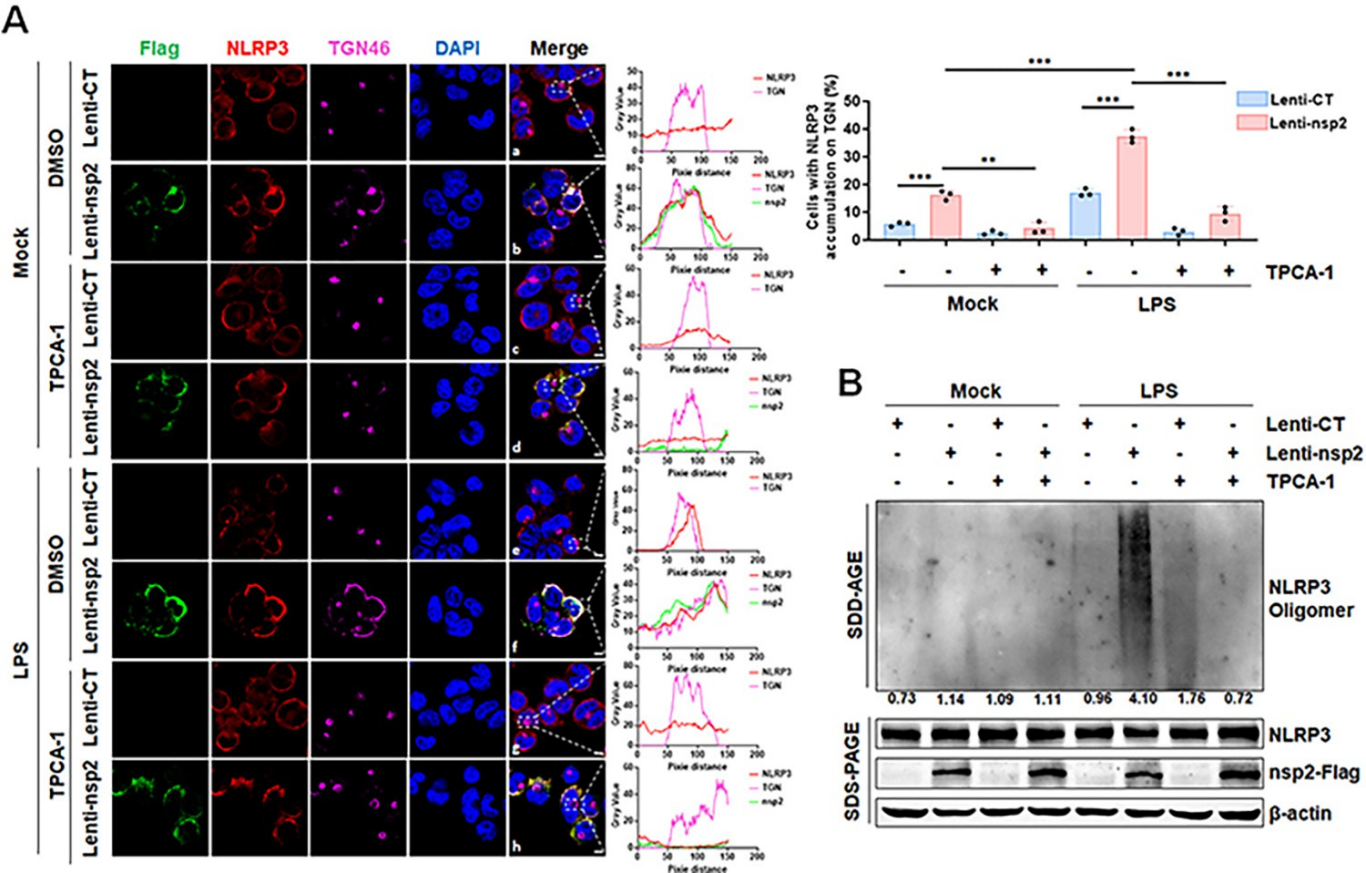
PRRSV-2 nsp2 protein promotes NLRP3 translocation to dTGN and oligomerization through IKKβ. (A and B) THP-1 cells stably infected with Lentivirus-CT or Lentivirus-nsp2, differentiated with PMA, pre-treated with 2.5 μM TPCA-1 or DMSO for 1 h, and then stimulated with 1 μg/mL LPS or medium for 12 h. Confocal images showing nsp2, NLRP3, and TGN46 co-localization are presented (A, left), with gray values measured by ImageJ (A, middle) and co-localization percentages shown (A, right). Cell lysates were analyzed by SDD-AGE and SDS-PAGE (B). Lenti, Lentivirus; CT, Control. For confocal microscopy assay, results were manually quantified from at least 30 randomly selected cells per condition per replicate using Image J. Scale bar: 10 μm. The protein levels were quantified by Image J and normalized to β-actin. The data are representative of results from three independent experiments. Error bars indicate the mean (± SD) of three repeats. *p ≤ 0.05, **p ≤ 0.01, ***p ≤ 0.001; p > 0.05 stands for ns.

Upon NLRP3 activation, the TGN disassembles and subsequently serves as a scaffold for NLRP3 oligomerization, initiating downstream signaling [32]. Therefore, we hypothesized that the nsp2 promotes NLRP3 translocation to the dTGN via IKKβ. Here, confocal microscopy result showed that, in the mock group, endogenous NLRP3 was diffusely distributed in the cytoplasm, while the TGN remained as a compact perinuclear cluster (Fig 7A (a)). In the presence of nsp2 protein, around 15% of NLRP3 translocated to the TGN, causing partial TGN disassembly into dispersed vesicles (Fig 7A (b)). However, TPCA-1 treatment effectively inhibited both NLRP3 translocation to the TGN and TGN disassembly (Fig 7A (d)). In the LPS stimulation group, about 15% of NLRP3 localized to the TGN (Fig 7A (e)). However, with nsp2 protein, approximately 35% of NLRP3 translocated to the TGN, leading to substantial TGN disassembly (Fig 7A (f)). Moreover, TPCA-1 exhibited similar functionality to the mock group (Fig 7A (g and h)). Additionally, SDD-AGE analysis revealed that TPCA-1 inhibitor markedly attenuated the NLRP3 oligomerization induced by nsp2 (Fig 7B). Collectively, these results indicated that PRRSV-2 nsp2 facilitates NLRP3 translocation to the dTGN for oligomerization via IKKβ.

### PRRSV-2 infection triggers inflammatory responses via IKKβ-dependent NLRP3 translocation to dTGN

**Fig 8:**
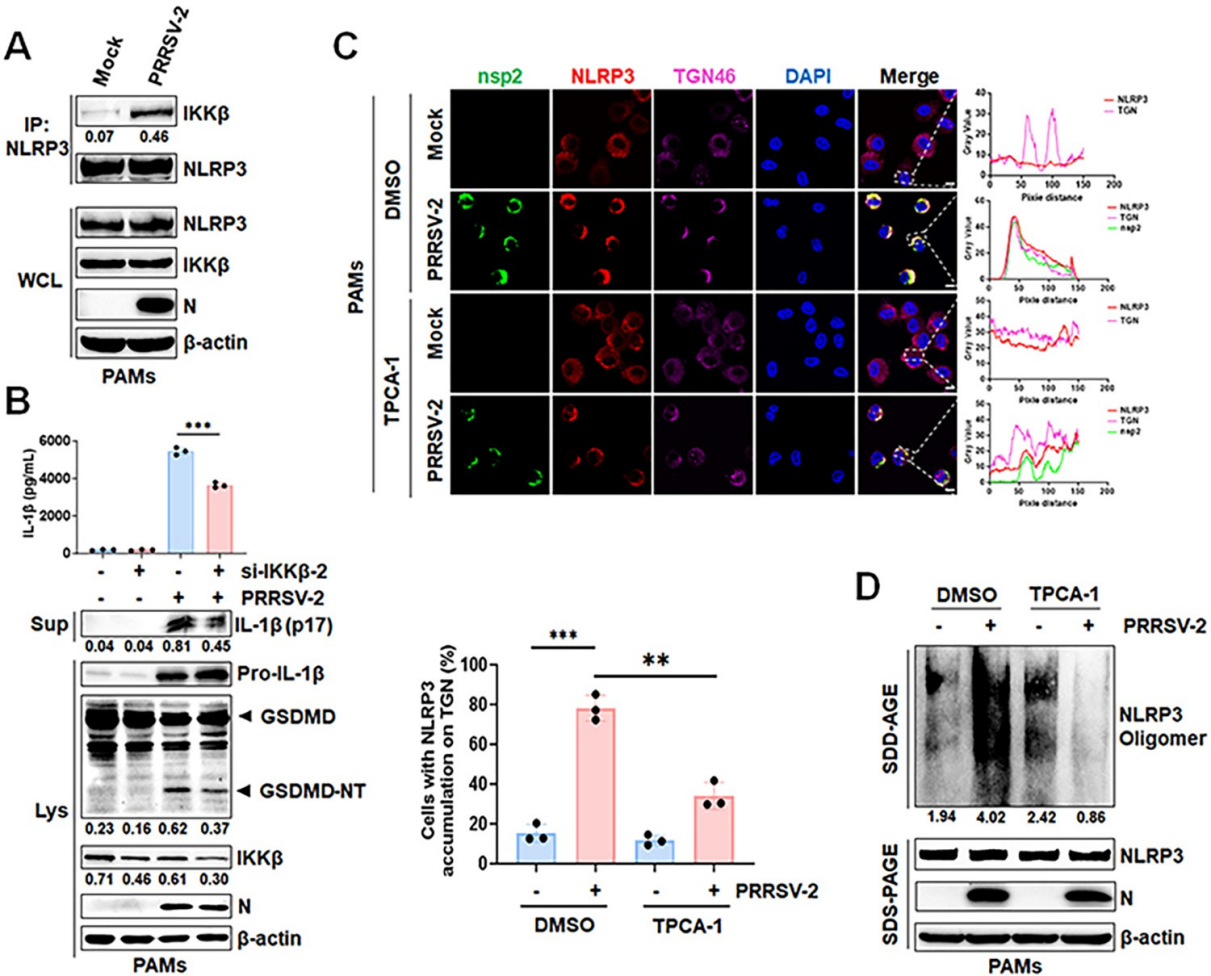
PRRSV-2 infection triggers inflammatory responses via IKKβ-dependent NLRP3 translocation to dTGN. (A) PAMs infected with PRRSV-2 (MOI of 0.05) for 18 h were immunoprecipitated with an anti-NLRP3 antibody. (B) PAMs transfected with control siRNA or siRNA targeting IKKβ (si-IKKβ-2) were infected with PRRSV-2 (MOI of 0.05) for 18 hours. IL-1β levels in supernatants were measured by ELISA, and mature IL-1β and GSDMD were detected by Western blot. (C and D) PAMs pre-treated with 2.5 μM TPCA-1 or DMSO for 1 h were infected with PRRSV-2 (MOI of 0.05). After 6 hpi, the medium was replaced with TPCA-1-free medium for another 6 h. Representative immunofluorescence images showing nsp2, NLRP3, and TGN46 co-localization were presented on (C, left). The gray value of each channel was measured by ImageJ software (C, right). The percentages of cells with NLRP3 and TGN46 co-localization were displayed on (C, bottom). Cell lysates were analyzed by SDD-AGE and SDS-PAGE (D). WCL, whole cell lysate; Lys, lysate; Sup, supernatant. For confocal microscopy assay, results were manually quantified from at least 30 randomly selected cells per condition per replicate using Image J. Scale bar: 10 μm. The protein levels were quantified by Image J and normalized to β-actin. The data are representative of results from three independent experiments. Error bars indicate the mean (± SD) of three repeats. *p ≤ 0.05, **p ≤ 0.01, ***p ≤ 0.001; p > 0.05 stands for ns.

As illustrated above, nsp2 facilitates NLRP3 inflammasome activation by regulating NLRP3 translocation to the dTGN and oligomerization via IKKβ. Therefore, we hypothesized that PRRSV-2 triggers an inflammatory response through a similar mechanism. Primally, Endogenous IP result revealed that PRRSV-2 infection in PAMs enhances the interaction between NLRP3 and IKKβ (Fig 8A). Moreover, downregulation of IKKβ expression via RNA interference and inhibited its activity with TPCA-1 markedly attenuated PRRSV-2-induced GSDMD cleavage and mature IL-1β secretion (Figs 8B, S1E and S4B). Additionally, confocal microscopy result revealed that upon PRRSV-2 infection, approximately 75% of NLRP3 translocated to the TGN, leading to substantial TGN disassembly. However, TPCA-1, an inhibitor of IKKβ, significantly attenuates PRRSV-2-induced translocation of NLRP3 to the dTGN (Fig 8C). Finally, SDD-AGE analysis revealed that PRRSV-2 infection promotes NLRP3 oligomerization, which was attenuated by inhibiting IKKβ activity (Fig 8D). Taken together, these results indicated that PRRSV-2 infection requires IKKβ-dependent NLRP3 translocation to dTGN for pyroptosis and inflammatory responses.

### IKKβ-dependent translocation of NLRP3 to the dTGN is essential for virus-induced inflammation

**Fig 9:**
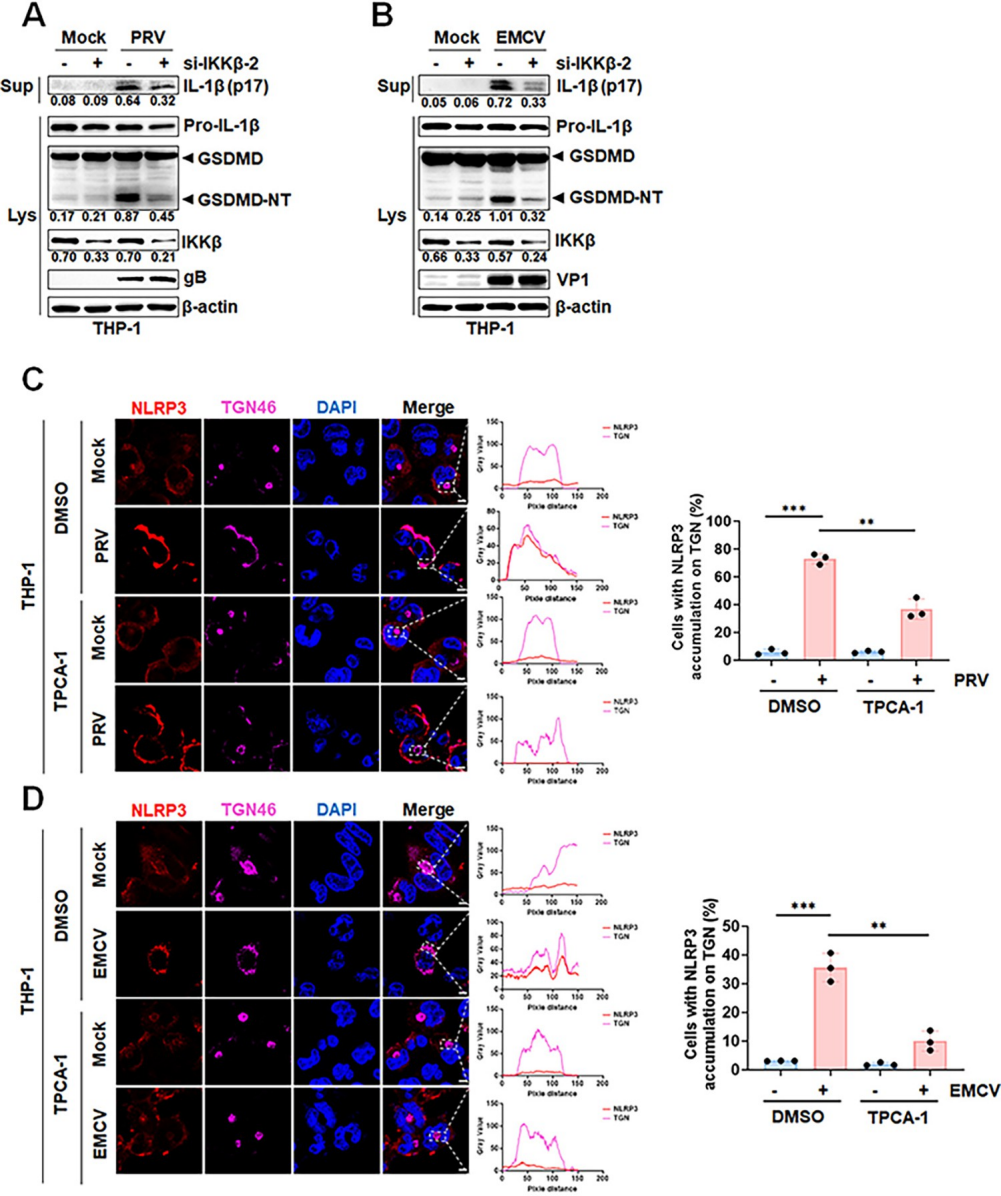
IKKβ-dependent translocation of NLRP3 to the dTGN is essential for virus-induced inflammation. (A and B) THP-1 cells were differentiated into macrophages using 100 ng/mL PMA. The cells were then transfected with either control siRNA (si-NC) or IKKβ-targeting siRNA (si-IKKβ-2) at a concentration of 100 nM for 24 hours, followed by infection with PRV (MOI of 5) (A) or EMCV (MOI of 10) for 12 hours (B). Western blot analysis was performed to detect mature IL-1β and GSDMD. (C and D) THP-1 cells differentiated into macrophages with 100 ng/mL PMA were pre-treated with either 2.5 μM TPCA-1 or DMSO for 1 hour before being infected with PRV (MOI of 5) (C) or EMCV (MOI of 10) for 12 hours (D). Representative immunofluorescence images captured via confocal microscopy showing co-localization of NLRP3 and TGN46 are displayed on the left. Gray values for each channel were quantified using ImageJ software (middle), and the percentage of cells with NLRP3 and TGN46 co-localization is shown on the right. Lys, lysate; Sup, supernatant. For confocal microscopy assay, results were manually quantified from at least 30 randomly selected cells per condition per replicate using Image J. Scale bar: 10 μm. The protein levels were quantified by Image J and normalized to β-actin. The data are representative of results from three independent experiments. Error bars indicate the mean (± SD) of three repeats. *p ≤ 0.05, **p ≤ 0.01, ***p ≤ 0.001; p > 0.05 stands for ns.

To investigate whether the IKKβ-mediated NLRP3 activation plays a role in virus-induced inflammation, both PRV and EMCV, which are known to induce NLRP3 inflammasome activation [33,34], were tested. We suppressed IKKβ expression via RNA interference and inhibited its activity with TPCA-1, resulting in a marked reduction in IL-1β production and GSDMD cleavage during PRV and EMCV infections (Figs 9A, 9B, S4C and S4D). Consistently, confocal microscopy results revealed that upon PRV and EMCV infection, the TGN disassembled from a single perinuclear cluster into dTGN, where NLRP3 accumulated. However, TPCA-1 prevented this process (Fig 9C and 9D). Therefore, these results suggested that viral infections, which triggered the NLRP3 inflammasome activation, required IKKβ-dependent translocation of NLRP3 to dTGN to promote its activation. In summary, the IKKβ-dependent translocation of NLRP3 to the dTGN plays a crucial role in viruses-induced inflammatory responses.

## Discussion

**Fig 10:**
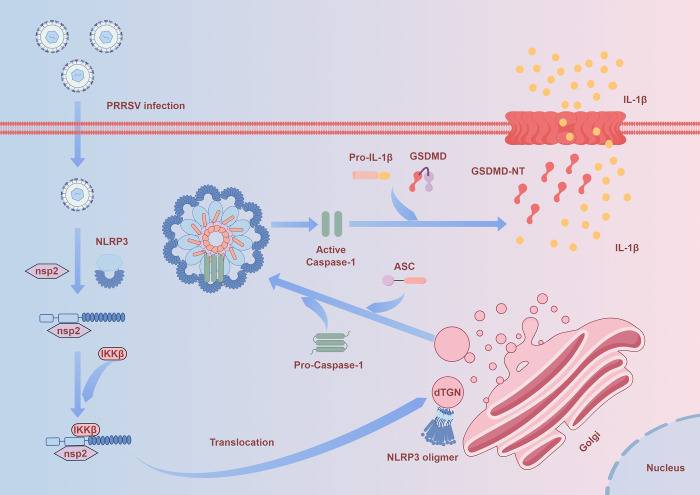
Schematic model of PRRSV-2 nsp2 protein activates NLRP3 inflammasome through IKKβ-dependent trans-Golgi network translocation. PRRSV-2 infection triggers inflammatory responses through the NLRP3 inflammasome. PRRSV-2 nsp2 interacts with NLRP3, facilitating the recruitment of IKKβ to NLRP3. This interaction ultimately promotes NLRP3 translocation to the dTGN and enhances its oligomerization in an IKKβ-dependent manner. Oligomerized NLRP3 further recruits ASC and pro-Caspase-1, forming the inflammasome complex and activating Caspase-1. Activated Caspase-1 then cleaves pro-IL-1β and GSDMD, triggering pyroptosis and the release of IL-1β, which ultimately results in pulmonary inflammatory injury. This picture was created by Figdraw (www.figdraw.com).

Excessive activation of NLRP3 inflammasome triggers intense inflammation, leading to severe tissue damage under various infections and stress signals [35–37]. Thus NLRP3 received much attention as a major regulator of antibacterial and sterile inflammation [38,39]. PRRSV-2 infection triggers NLRP3 inflammasome activation and induces pyroptosis [16,18]. But the mechanism remains insufficiently understood. In this study, PRRSV-2 nsp2 was identified as a key ignitor for NLRP3 inflammasome activation. Mechanistically, the nsp2 interacts with NLRP3 to promote the recruitment of IKKβ, facilitating the translocation of NLRP3 to the dTGN. At the dTGN, NLRP3 undergoes oligomerization and recruits additional ASC proteins to complete the inflammasome assembly process (Fig 10). It provides valuable insights into PRRSV-2 pathogenesis.

Some viruses, including EMCV, vesicular stomatitis virus (VSV), and PRV, activate NLRP3 by promoting potassium efflux via lytic cell death [33,34]. Alternatively, viral nucleic acids [40] or proteins, such as the Zika virus NS5 protein [41], dengue virus M protein [42], SARS-CoV-2 nucleocapsid protein [43], and Enterovirus 71 (EV71) 3D nonstructural protein [44] could also activate NLRP3, either directly or indirectly. Here, our screening revealed that PRRSV-2 nsp2, nsp4, and nsp5 induce IL-1β secretion in the HEK293T-NLRP3 inflammasome system, with nsp2 showing the strongest activity. Nsp2 is a pivotal multifunctional protein in PRRSVs, intricately involved in viral assembly, innate immunity suppression, autophagy induction, and viral virulence, and it contains both B-cell and predicted T-cell epitopes [45]. With its potent IL-1β induction and close link to PRRSVs virulence, nsp2 was chosen as our primary focus. Nsp4, the viral main protease, exhibits 3C-like protease (3CLP) activity to cleave viral polyproteins and host factors involved in the innate immune responses [46,47]. Nsp5, a multi-transmembrane protein, plays a role in autophagosome formation, inhibits autophagosome-lysosome fusion, and antagonizes host innate immunity [48–50]. Both nsp4 and nsp5 may also contribute to NLRP3 inflammasome activation, although the underlying molecular mechanisms need to be further studied.

The canonical NLRP3 inflammasome activation typically needs both a priming signal (e.g., LPS, signal 1) and an activating signal (e.g., nigericin, signal 2) [3]. To elucidate the specific stage of NLRP3 inflammasome activation influenced by PRRSV-2 nsp2, we used THP-1 cells, a widely utilized human monocytic cell line in inflammation research [29,51,52]. Our results showed that nsp2, in conjunction with LPS, induces IL-1β secretion and pyroptosis, indicating its primarily functions during the activation stage. Furthermore, nsp2 interacts with NLRP3 via its TM domain, promoting NLRP3 inflammasome assembly. It indicates that nsp2 induces canonical NLRP3 inflammasome activation. Usually, the non-canonical inflammasomes are triggered by intracellular LPS, recognized by mouse caspase-11 or human caspase-4/5 to trigger pyroptosis via GSDMD cleavage [53]. Further studies are needed to determine whether nsp2 can activate non-canonical inflammasome pathways.

NEK7 is a classical regulator of NLRP3 and plays an important role in inflammasome activation [54]. Interestingly, Schmacke and colleagues reported a NEK7-independent pathway within the primary activation of NLRP3 in human cells where IKKβ replaced the role of NEK7 [29]. In this study, we observed that NEK7 downregulation did not impede nsp2-mediated IL-1β secretion and pyroptosis. However, suppressing IKKβ expression or inhibiting its activity significantly reduced PRRSV-2 or nsp2-induced inflammatory responses. During NLRP3 activation, NLRP3 got oligomerized in dTGN to form an active structure, further promoting NLRP3 inflammasome activation [32]. Here, we found that both PRRSV-2 and nsp2 facilitate IKKβ recruitment to NLRP3, enhancing NLRP3 translocation to the TGN and driving TGN disassembly, ultimately inducing NLRP3 oligomerization. However, inhibition of IKKβ activity effectively disrupts this process. In addition, it was also found that the IKKβ-dependent translocation of NLRP3 to the dTGN is critical for the inflammatory responses induced by PRV and EMCV. Therefore, these results underscore the important role of IKKβ in virus-mediated inflammatory responses.

The IKKβ component of the canonical IKK complex plays multiple roles in innate immunity, cell metabolism, and disease. Through its kinase activity, it activates key transcription factors such as NF-κB and interferon regulatory factor 5 (IRF5), as well as other protein kinases, including the IKK-related kinases TBK1 (TANK-binding kinase 1) and IKKɛ [11,55,56]. In this study, our results showed that the N-terminal kinase domain of IKKβ interacts with the NACHT and LRR domains of NLRP3. However, whether IKKβ activates NLRP3 through its kinase activity remains to be determined in future studies.

ASC serves as an inflammasome adaptor protein, and its oligomerization is a hallmark of inflammasome activation [3]. After inflammasome activation, ASC assembles into a large protein complex, which is termed "speck" [57]. Here, we observed multiple ASC specks in PAMs following PRRSV-2 infection or LPS plus NG control, differing from the typical speck that forms a single speck per cell. Since the ASC oligomerization and other readouts of inflammasome activation are apparent, it is difficult to assess if these ASC speckles are not fully formed inflammasomes or if inflammasome formation in PAMs is simply different from what is seen in humans and mice. It remains to be elucidated.

PRRSV-2 nsp2 comprises a cysteine protease (PLP2), a central hypervariable (HV), a transmembrane (TM) and a C-terminal tail domains. The PLP2 domain exhibits cis and trans cleavage activities, the flexible HV domain contains multiple B-cell epitopes, and the C-terminal tail plays a crucial role in aggrephagy by interacting with cellular protein 14-3-3ε [27,45,58]. Meanwhile, aggresome is important for NLRP3 inflammasome activation, and the dynein adapter histone deacetylase 6 (HDAC6) plays a crucial role during this process [59]. Here, it is interesting that only the TM domain, containing C-terminal tail is sufficient to activate the NLRP3 inflammasome. Whether the TM domain promotes NLRP3 inflammasome activation through the formation of aggresome remains to be elucidated.

In addition, we noted that knockdown the expression of NLRP3 or inhibitor MCC950 just slightly restore PRRSV-2 infection-induced expression of the tested cytokines and chemokines. It suggested that other critical NLRP, such as NLRP1, may also involve in PRRSV-2 induced inflammatory responses, which needing further investigation. By the way, in this study, we screened and detected the nsp2-mediated activation of the NLRP3 inflammasome using human-derived cells, but not porcine cell lines (Figs 2 and 3), since the low transfection efficiency of the recombinant plasmids, which is a limitation of this study.

Collectively, in this study, we discovered a novel mechanism of NLRP3 inflammasome activation during PRRSV-2 infection. PRRSV-2 employs nsp2 to facilitate NLRP3 translocation to the dTGN, ultimately inducing NLRP3 oligomerization, with IKKβ serving as a pivotal mediator. These findings advance our understanding of PRRSV-2 pathogenesis and underscore the critical role of IKKβ in virus-mediated inflammatory responses.

## Materials and methods

### Ethics statement

The animal experiments conformed to the rules of National Guidelines for Housing and Care of Laboratory Animals (China) and were performed after obtaining the approval of the Institutional Animal Care and Ethics Committee of Nanjing Agricultural University (permit no. IACECNAU20191002).

### Cell lines and cultures

Human embryonic kidney cell line (HEK293T) and the human monocytic THP-1 cell line were purchased from the American Type Culture Collection (ATCC). HEK293T cells were cultured in Dulbecco’s Modified Eagle Medium (DMEM, Gibco). THP-1 cells were cultured in RPMI 1640 medium (Gibco). Porcine alveolar macrophages (PAMs) were collected from 4-week-old, specific-pathogen-free piglets as previously described and grown in RPMI 1640 medium [60]. All cells were supplemented with 10% fetal bovine serum, 100 U/mL penicillin, and 100 μg/mL streptomycin, and maintained at 37°C in a 5% CO_2_ incubator. THP-1 cells were differentiated into macrophages by stimulating with 100 ng/mL PMA for 12 h, followed by culturing in complete medium for 24 h before stimulation or infection.

### Viruses

North American genotype 2 PRRSV strain BB0907 (GenBank accession no. HQ315835.1), The EMCV strain NJ08 (GenBank accession no. HM641897), and PRV strain ZJ01 (GenBank accession no. KM061380.1) were used for this investigation and referred to as PRRSV-2, EMCV, and PRV, respectively. Viruses’ propagation was performed as described previously [61–63].

### Reagents and antibodies

Swine IL-1β ELISA kit (CSE0013) was purchased from 4A Biotech, China. Nigericin (N7143), LPS (L3024), dansylsarcosine piperidinium salt (DSS) (S1885) and anti-Flag M2 magnetic beads (M8823) were purchased from Sigma-Aldrich. Lipofectamine 3000 reagent (L3000015) and Lipofectamin RNAiMAX Transfection Reagent (13778075) were purchased from Invitrogen. Total RNA Kit I (R6834) was purchased from Omega Bio-Tek. Protein A+G Agarose (P2012) was purchased from Beyotime. EasyCap T7 Cotranscription Kit with CAG Trimer (DD4203-01), VAHTS RNA Clean Beads (N412-01), HiScript II 1st Strand cDNA Synthesis Kit (R211-01), and AceQ qPCR SYBR Green Master Mix (Q111-02) were purchased from Vazyme. MCC950 (S7809), Z-YVAD-FMK (S8507), PMA (S7791), puromycin (S7417), and TPCA-1 (S2824) were purchased from Selleck.

Antibodies in this paper were listed as follows: mouse anti-PRRSV-2-N protein, mouse anti-PRRSV-2-nsp2 protein, mouse anti-EMCV-VP1 protein, and mouse anti-PRV-gB protein were produced in our laboratory. Rabbit anti-NLRP3 (27458-1-AP), rabbit anti-ASC (10500-1-AP), rabbit anti-IKKβ (15649-1-AP), mouse anti-β-actin (66009-1-Ig), CoraLite488-conjugated Goat Anti-Mouse IgG (SA00013-1), CoraLite594-conjugated Goat Anti-Rabbit IgG(H+L) (SA00013-4) and Rhodamine (TRITC)-conjugated Donkey Anti-Goat IgG(H+L) (SA00007-3) were purchased from Proteintech. Goat Anti-Rabbit IgG (H+L) Fluor647-conjugated (S0013) was purchased from Affinity. Goat anti-NLRP3 (ab4207) was purchased from Abcam. Goat anti-IL-1β (NB600-633), and rabbit anti-GSDMD (NBP2-33422) were purchased from Novus. Rabbit anti-IL-1β (12703S) was purchased from CST. Rabbit anti-NEK7 (A19816) was purchased from ABclonal. Rabbit anti-HA Tag (R20003), mouse anti-HA Tag (M20003), rabbit anti-Flag Tag (R20008), mouse anti-Flag Tag (M20008), rabbit anti-Myc Tag (R20002), and mouse anti-Myc Tag (M20002) were purchased from Abmart. Rabbit IgG (A7016), mouse IgG (A7028), and horseradish peroxidase (HRP)-labeled rabbit (A0208) or mouse (A0216) secondary antibodies were purchased from Beyotime.

### RNA extract and qRT-PCR

Total RNAs were extracted by using Total RNA Kit I according to the manufacturer’s instructions; the RNA was then reverse-transcribed into cDNA by using HiScript II 1st Strand cDNA Synthesis Kit. qRT-PCR was performed using the AceQ qPCR SYBR Green Master Mix on QuantStudio 5 (Applied Biosystems, USA). The qRT-PCR primers were designed by Primer 3 Plus, and listed in S1 Table. The qRT-PCR data were normalized by the level of β–actin expression. 2^^(-ΔΔCt)^ method was used to calculate relative expression changes.

### In vitro transcription and transfection

For overexpression of eGFP and eGFP-TM on PAMs, the mRNA of eGFP and eGFP-TM genes were prepared by using a series of in vitro transcription kits and transfected using Lipofectamine 3000 (Invitrogen) according to the manufacturer’s instructions.

### Plasmid construction and transfection

Plasmids encoding Flag-tagged PRRSV-2 structural and non-structural proteins were constructed previously by our laboratory [64]. The genes for NLRP3 (GenBank accession no. AB292177.1), ASC (GenBank accession no. AB873106.1), Caspase-1 (GenBank accession no. NM_214162.1), IL-1β (GenBank accession no. NM_214055.1), and IKKβ (GenBank accession no. NM_001099935.1) amplified from PAMs, were inserted into pCAGGS with a 3’ Flag, HA or Myc tag. For the truncated forms of PRRSV-2 nsp2, NLRP3, and IKKβ the PCR productions were inserted into pCAGGS. The PCR primers were listed in S2 Table. All the plasmid constructs were confirmed by DNA Sanger sequencing. These expression plasmids were transfected into HEK293T cells by using PEI (Sigma) according to the manufacturer’s instructions.

### Reconstituted NLRP3 inflammasome in HEK293T cells

The NLRP3 inflammasome reconstitute assay in HEK293T cells was referred to previous papers with some modification [43]. Briefly, HEK293T cells were transfected with NLRP3 (200 ng), pro-Caspase-1 (100 ng), ASC (100 ng), or pro-IL-1β (200 ng), and then transfected with plasmids expressing indicated proteins for 24 h. Cell supernatants were collected and analyzed for IL-1β secretion level by ELISA assay and IL-1β maturity level by Western blotting, and cells were lysed and proteins in the cell analyzed by Western blotting.

### Small interfering RNA (siRNA) transfection

For knockdown experiments, the small interfering RNA (siRNA) of targeted genes were obtained from Genepharma (Shanghai, China) and transfected into PAMs or THP-1 cells for 24 h using Lipofectamine RNAiMAX according to the manufacturer’s instructions. The siRNAs used in this study are presented in S3 Table.

### Lentivirus-mediated gene expression

For stable expression of the nsp2 protein in THP-1 cells, lentiviral transduction was performed as described previously [43]. Briefly, lentiviruses were produced after transfection of pCDH-nsp2-Flag or pCDH-Flag empty vector together with packaging plasmids pSPAX2 and pMD2G with a ratio of 4:3:1 in HEK293T cells. The lentivirus packaged with pCDH-nsp2-Flag was named Lentivirus-nsp2, and the lentivirus packaged with pCDH-Flag was named Lentivirus-Control (CT). The supernatants were collected 72 h after transfection. Then THP-1 cells were infected with the lentivirus. At 72 h after infection, the cells were selected with 3 μg/mL puromycin for 2 days. All experiments were performed within 2 weeks after lentiviral transduction.

### Enzyme-linked immunosorbent assay (ELISA)

The concentration of IL-1β in cell culture supernatant was measured by an ELISA Kit according to the manufacturer’s instructions. Briefly, a 50 μL sample was added to the ELISA well, incubated for 1.5 h at 37°C, and then aspirated and washed five times. Anti- IL-1β antibody (100 μL) was added to each well, incubated for 1 h at 37°C, and aspirated and washed five times. Enzyme (100 μL) was added to each well, incubated for 30 min at 37°C, and aspirated and washed five times. 3,3’,5,5’tetramethylbenzidine (TMB) one-step substrate reagent (100 μL) was added to each well and incubated for 15 min at 37°C. Stop solution (100 μL) was added to each well. The absorbance was measured at 450 nm.

### Western blotting

Cells were dissolved in lysis buffer (20 mM Tris [pH 7.5], 150 mM NaCl, 1% Triton X-100, sodium pyrophosphate, b-glycerophosphate, EDTA, Na_3_VO_4_, and leupeptin). Protein concentration was measured by BCA assay (Vazyme, China). Cell lysates (40 μg) were resolved on 8–12% SDS–PAGE gels and transferred onto the PVDF membrane (EMD Millipore, USA). Membranes were blocked in 5% (wt/vol) nonfat milk in TBST (20 mM Tris–HCl, pH 7.6, 150 mM NaCl, and 0.1% (vol/vol) Tween-20) for 2 h at room temperature. Membranes were probed with primary antibody diluted in 3% (wt/vol) BSA in TBST at 4°C overnight. The membrane was washed three times for 10 min with TBST and then incubated with corresponding secondary antibodies for 1 h at room temperature. Protein bands were visualized using a gel imaging system (Tanon, China).

### Co-immunoprecipitation assays (Co-IP)

Immunoprecipitation was performed as described previously [65]. Cells were grown to 70–80% confluence in 10cm dishes and transfected or stimulated as indicated. Then, the cells were dissolved in lysis buffer rotated at 4°C for 30 min, and centrifuged at 8000 × g for 10 min to remove debris. 100 μL of supernatant were used as input, and the rest of the supernatants were incubated with anti-Flag M2 magnetic beads or the indicated antibodies plus Protein A/G beads overnight at 4°C. Immunoprecipitates were washed five times with low-salt lysis buffer and resuspended in 60 μL 2 x SDS loading buffer and boiled at 100°C for 10 min and analyzed by western blotting.

### Confocal microscopy

Cells were cultured on glass bottom culture dishes (Nest Scientific, China) at a density of 5 × 10^5^/mL and transfected/treated as indicated. Cells were washed with ice-cold PBS three times and fixed with 4% paraformaldehyde (Biosharp, China) for 30 min at room temperature, then permeabilized with PBS containing 0.1% Triton X-100 for 10 min. After washing with PBS three times, the cells were blocked with PBS containing 2% BSA (blocking buffer) for 1 h and incubated with the indicated primary antibodies diluted in blocking buffer at 4°C overnight. After washing with PBS three times, the cells were incubated with a fluorescently labeled secondary antibody for 1 h at room temperature in the dark. Nuclei were stained with DAPI (4′,6-diamidino-2-phenylindole) for 10 min, and the cells were washed three times. To better preserve the dTGN structures in fixed cells, we substituted 0.1% saponin for 0.1% Triton X-100 in the permeabilization step. Fluorescence was observed by confocal microscopy (LSM800 microscope; Zeiss).

### ASC oligomerization analysis

Cells were lysed in lysis buffer, incubated at 4°C for 30 min with gentle shaking, and then centrifuged at 5000 × g at 4°C for 10 min. The pellets were washed with PBS, re-suspended in 500 μL PBS, and treated with 2 mM DSS for cross-linking at 37°C for 30 min. After centrifugation at 4000 × g for 10 min, the supernatant was discarded, and the cross-linked pellets were re-suspended in 50 μL 2 × SDS loading buffer. The samples were boiled for 10 min and then analyzed by western blotting.

### Semidenaturing detergent agarose gel electrophoresis (SDD-AGE)

SDD-AGE was performed according to a published protocol with minor modifications [66]. In brief, cells were either infected with PRRSV-2 or stimulated with LPS for the specified duration. Following treatment, cells were lysed with 1 × SDD sample buffer (1 × TBE buffer, 10% Glycerol, 2% SDS, 25% Bromophenol blue). NLRP3 oligomers were separated by 1.5% agarose gel in SDD-AGE running buffer (1 × TBE and 0.1% SDS) for 80 min with 100 V at 4°C. Subsequently, samples were transferred to PVDF membranes (Millipore) and probed using an anti-NLRP3 antibody for detection.

### Statistical analyses

All experiments were repeated at least three times. The data of all quantitative experiments are represented as the mean ± SD. The gray value of fluorescence in images was analyzed by ImageJ 1.8.0 software (National Institutes of Health). Student’s t-test was used to compare differences between two groups for normally distributed data. The p values were calculated from three biological replicates and considered statistically significant when *p ≤ 0.05, **p ≤ 0.01, ***p ≤ 0.001; p > 0.05 stands for not significant (ns). All quantitative data were analyzed using GraphPad Prism 8 (GraphPad Software, inc. USA).

## Supporting information

S1 Fig Evaluation of siRNA effectiveness. (A) PAMs were transfected with control siRNA (si-NC) or NLRP3-targeting siRNAs (si-NLRP3-1, si-NLRP3-2, or si-NLRP3-3) at 100 nM for 24 h. NLRP3 protein levels were analyzed by Western blot. (B) THP-1 cells were transfected with si-NC or NLRP3-targeting siRNAs (si-NLRP3-1, si-NLRP3-2, or si-NLRP3-3) at 100 nM for 24 h. NLRP3 protein levels were analyzed by Western blot. (C) THP-1 cells were transfected with si-NC or NEK7-targeting siRNAs (si-NEK7-1, si-NEK7-2, or si-NEK7-3) at 100 nM for 24 h. NEK7 protein levels were analyzed by Western blot. (D) THP-1 cells were transfected with si-NC or IKKβ-targeting siRNAs (si-IKKβ-1, si-IKKβ-2, or si-IKKβ-3) at 100 nM for 24 h. IKKβ protein levels were analyzed by Western blot. (E) PAMs were transfected with si-NC or IKKβ-targeting siRNAs (si-IKKβ-1, si-IKKβ-2, or si-IKKβ-3) at 100 nM for 24 h. IKKβ protein levels were analyzed by Western blot.
(TIF)

S2 Fig The proinflammation responses induced by PRRSV-2 in PAMs pre-treated with MCC950. PAMs were pre-treated with 50 μM MCC950 for 1 hour, then infected with PRRSV-2 at an MOI of 0.05 for 18 hours. Gene transcription levels in PAMs were evaluated by qRT-PCR. The relative expression changes were calculated by using 2^^(-ΔΔCt)^ method. The data are representative of results from three independent experiments. Error bars indicate the mean (± SD) of three repeats. *p ≤ 0.05, **p ≤ 0.01, ***p ≤ 0.001; p > 0.05 stands for ns.
(TIF)

S3 Fig PRRSV-2 nsp2 promotes the activation of NLRP3 inflammasome. (A) HEK293T cells co-transfected with HA-tagged NLRP3, ASC, pro-CASP1, pro-IL-1β, and Flag-tagged PRRSV-2 protein plasmids or empty vector (pCAGGS) for 24 h. The protein expression was analyzed by Western blot. (B) THP-1 cells were stably infected with Lentivirus-CT or Lentivirus-Nsp2, differentiated into macrophages with 100 ng/mL PMA, and treated with 50 μM MCC950 for 12 h. They were then stimulated with 1mg/mL LPS or culture media for 12 h. Mature IL-1β (p17) in supernatants and GSDMD in lysates were analyzed by Western blot. Sup, supernatant; Lys, lysate. The protein levels were quantified by Image J and normalized to β-actin. The data are representative of results from three independent experiments.
(TIF)

S4 Fig IKKβ is essential for the inflammatory responses during viral infections. (A) THP-1 cells were stably infected with lentivirus-CT or lentivirus-Nsp2, differentiated into macrophages with 100 ng/mL PMA, and treated with 2.5 μM TPCA-1 for 1 h. They were then stimulated with 1mg/mL LPS or culture media for 12 h. Mature IL-1β (p17) in supernatants and GSDMD in lysates were analyzed by Western blot. (B) PAMs were pre-treated with 2.5 μM TPCA-1 or DMSO for 1 hour, then infected with PRRSV-2 at an MOI of 0.05. After 6 hpi, the culture medium was replaced with TPCA-1-free medium for an additional 12 h. IL-1β levels in the supernatants were determined by ELISA. Mature IL-1β (p17) in supernatants and GSDMD in lysates were determined by Western blot. (C and D) THP-1 cells were differentiated into macrophages using 100 ng/mL PMA. These cells were then pre-treated with either 2.5 μM TPCA-1 or DMSO for 1 h, followed by infection with PRV (MOI of 5) (C) or EMCV (MOI of 10) for 12 hours (D). Western blot analysis was performed to detect mature IL-1β and GSDMD. Sup, supernatant; Lys, lysate. The protein levels were quantified by Image J and normalized to β-actin. The data are representative of results from three independent experiments. Error bars indicate the mean (± SD) of three repeats. *p ≤ 0.05, **p ≤ 0.01, ***p ≤ 0.001; p > 0.05 stands for ns.
(TIF)

S1 Table Primers used for qRT-PCR. (XLSX)

S2 Table Primers used for PCR in this study. (XLSX)

S3 Table Oligonucleotides sequences of siRNA used in this study. (XLSX)

S4 Table Numerical data of this study. Excel spreadsheet containing the underlying numerical data for Figs 1A, 1B, 1C, 1D, 1E, 1F, 1I, 2A, 2B, 2C, 3D, 4C, 4D, 4E, 5C, 5D, 5E, 7A, 8B, 8C, 9C, 9D, S2, S4B in separate sheets.
(XLSX)
