# Supplementary figures and images for: PRRSV-2 nsp2 Ignites NLRP3 inflammasome through IKKβ-dependent dispersed trans-Golgi network translocation
Source: PLoS Pathog. 2025 Jan 27;21(1):e1012915. doi: 10.1371/journal.ppat.1012915 (PMC11801707; doi:10.1371/journal.ppat.1012915)

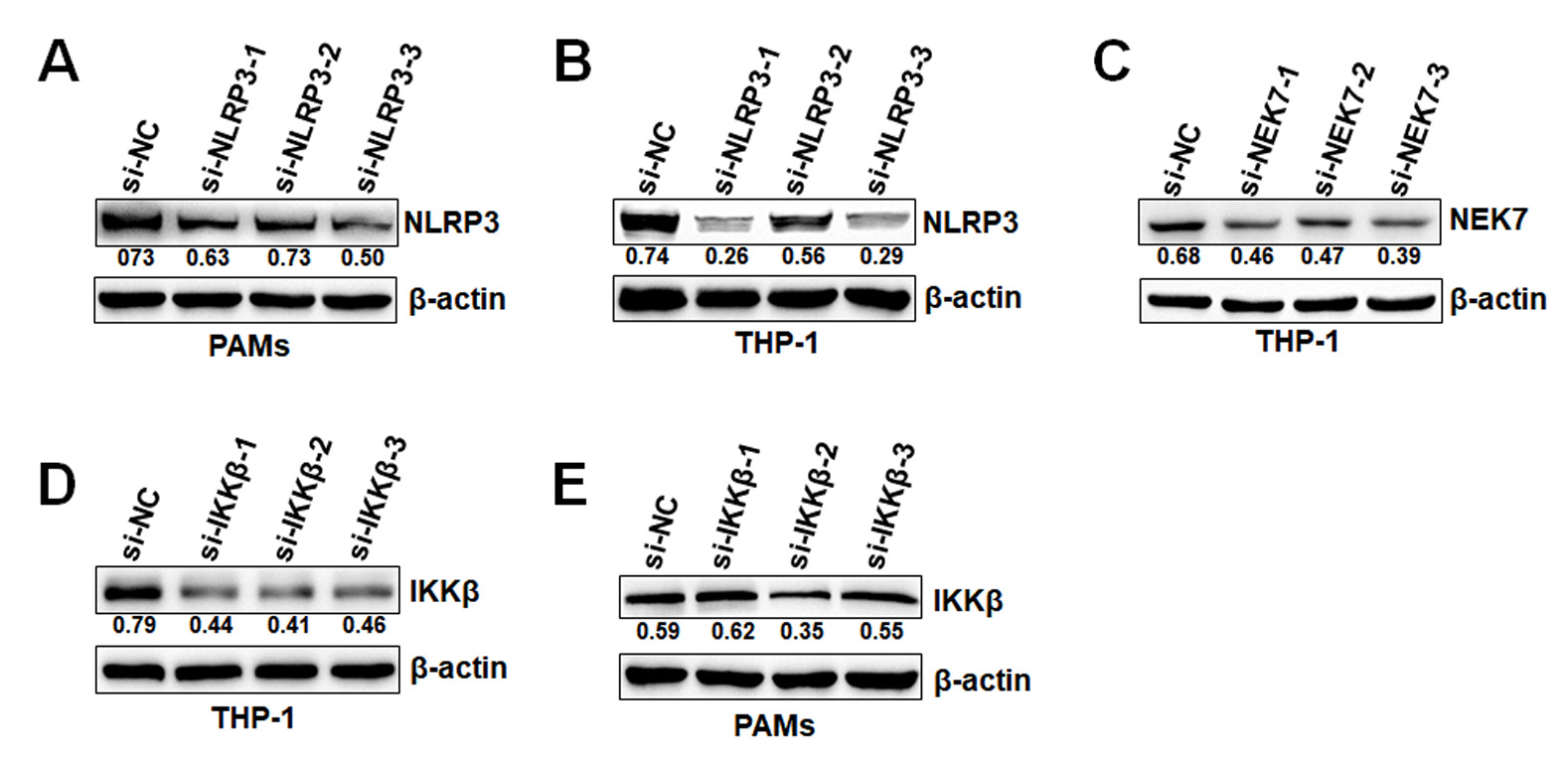

Supplement: S1 Fig — (A) PAMs were transfected with control siRNA (si-NC) or NLRP3-targeting siRNAs (si-NLRP3-1, si-NLRP3-2, or si-NLRP3-3) at 100 nM for 24 h. NLRP3 protein levels were analyzed by Western blot. (B) THP-1 cells were transfected with si-NC or NLRP3-targeting siRNAs (si-NLRP3-1, si-NLRP3-2, or si-NLRP3-3) at 100 nM for 24 h. NLRP3 protein levels were analyzed by Western blot. (C) THP-1 cells were transfected with si-NC or NEK7-targeting siRNAs (si-NEK7-1, si-NEK7-2, or si-NEK7-3) at 100 nM for 24 h. NEK7 protein levels were analyzed by Western blot. (D) THP-1 cells were transfected with si-NC or IKKβ-targeting siRNAs (si-IKKβ-1, si-IKKβ-2, or si-IKKβ-3) at 100 nM for 24 h. IKKβ protein levels were analyzed by Western blot. (E) PAMs were transfected with si-NC or IKKβ-targeting siRNAs (si-IKKβ-1, si-IKKβ-2, or si-IKKβ-3) at 100 nM for 24 h. IKKβ protein levels were analyzed by Western blot. (TIF) [file ppat.1012915.s001.tif]

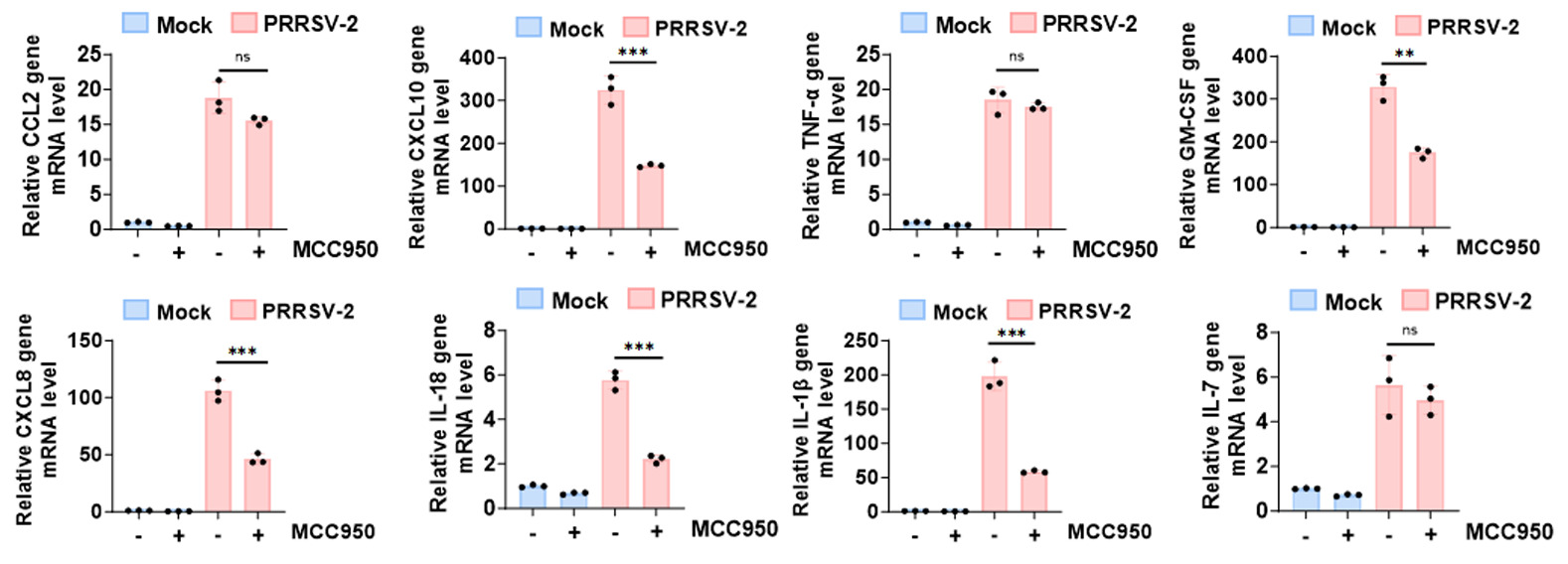

Supplement: S2 Fig — PAMs were pre-treated with 50 μM MCC950 for 1 hour, then infected with PRRSV-2 at an MOI of 0.05 for 18 hours. Gene transcription levels in PAMs were evaluated by qRT-PCR. The relative expression changes were calculated by using 2^(-ΔΔCt) method. The data are representative of results from three independent experiments. Error bars indicate the mean (± SD) of three repeats. *p ≤ 0.05, **p ≤ 0.01, ***p ≤ 0.001; p > 0.05 stands for ns. (TIF) [file ppat.1012915.s002.tif]

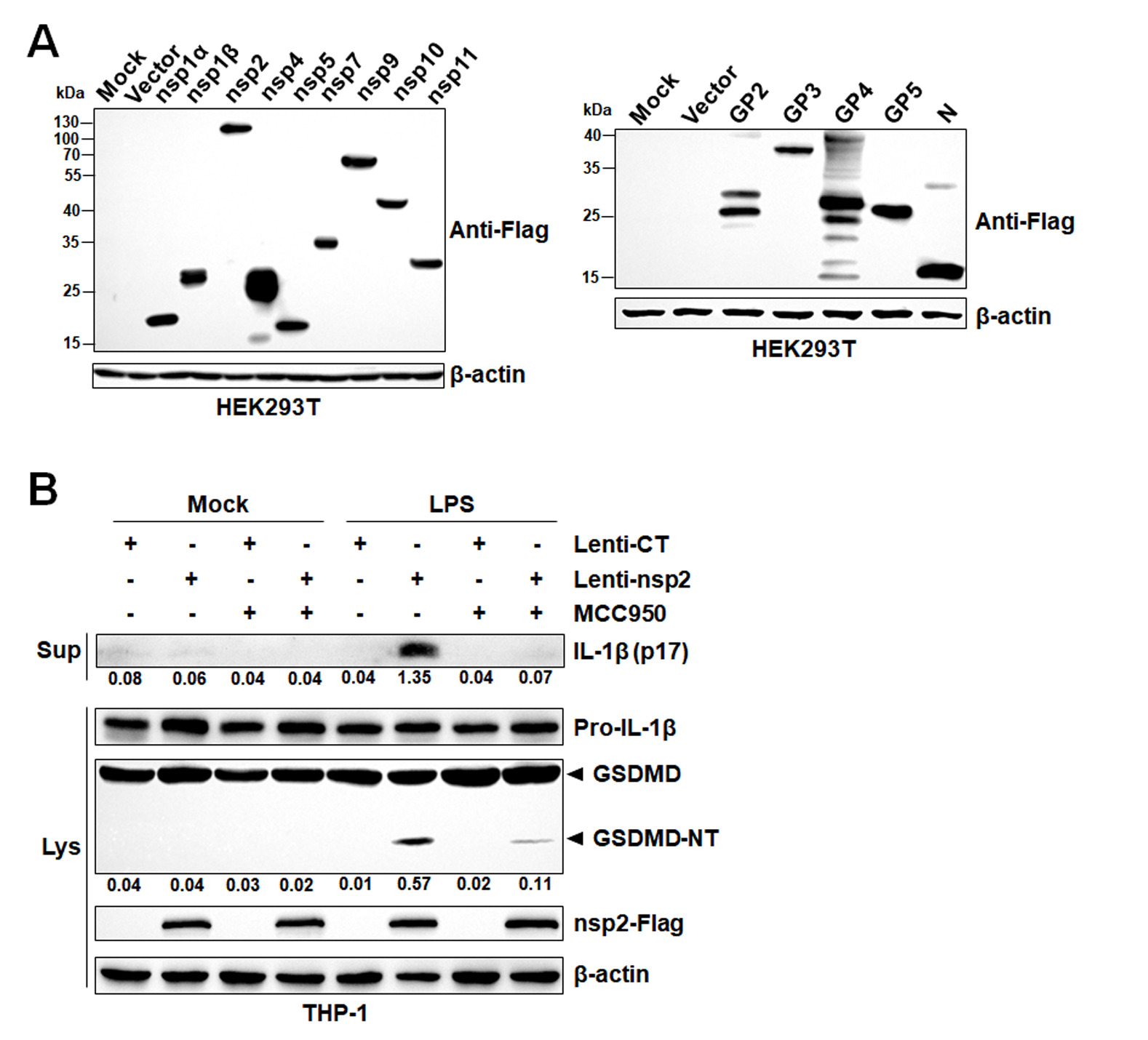

Supplement: S3 Fig — (A) HEK293T cells co-transfected with HA-tagged NLRP3, ASC, pro-CASP1, pro-IL-1β, and Flag-tagged PRRSV-2 protein plasmids or empty vector (pCAGGS) for 24 h. The protein expression was analyzed by Western blot. (B) THP-1 cells were stably infected with Lentivirus-CT or Lentivirus-Nsp2, differentiated into macrophages with 100 ng/mL PMA, and treated with 50 μM MCC950 for 12 h. They were then stimulated with 1mg/mL LPS or culture media for 12 h. Mature IL-1β (p17) in supernatants and GSDMD in lysates were analyzed by Western blot. Sup, supernatant; Lys, lysate. The protein levels were quantified by Image J and normalized to β-actin. The data are representative of results from three independent experiments. (TIF) [file ppat.1012915.s003.tif]

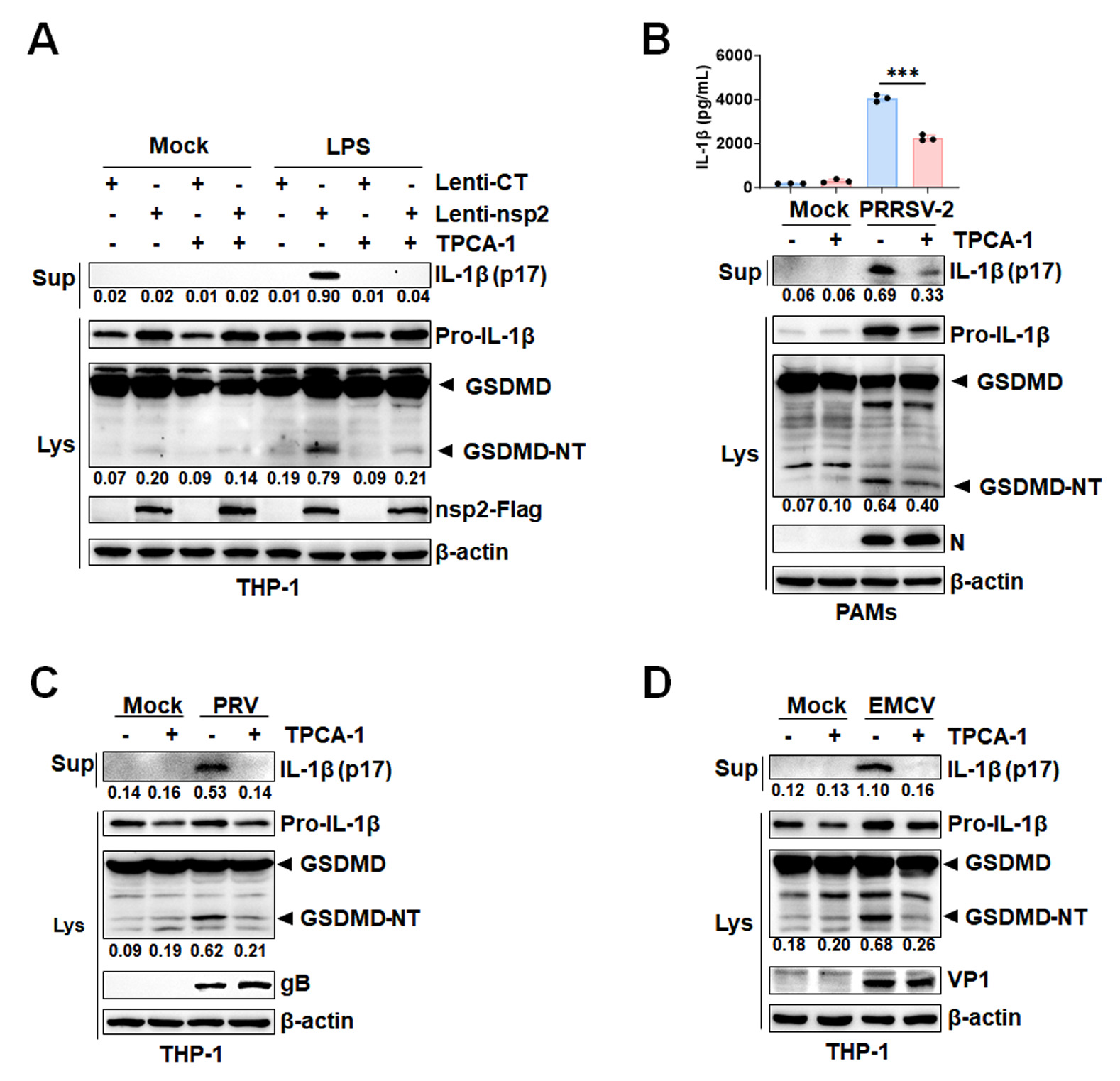

Supplement: S4 Fig — (A) THP-1 cells were stably infected with lentivirus-CT or lentivirus-Nsp2, differentiated into macrophages with 100 ng/mL PMA, and treated with 2.5 μM TPCA-1 for 1 h. They were then stimulated with 1mg/mL LPS or culture media for 12 h. Mature IL-1β (p17) in supernatants and GSDMD in lysates were analyzed by Western blot. (B) PAMs were pre-treated with 2.5 μM TPCA-1 or DMSO for 1 hour, then infected with PRRSV-2 at an MOI of 0.05. After 6 hpi, the culture medium was replaced with TPCA-1-free medium for an additional 12 h. IL-1β levels in the supernatants were determined by ELISA. Mature IL-1β (p17) in supernatants and GSDMD in lysates were determined by Western blot. (C and D) THP-1 cells were differentiated into macrophages using 100 ng/mL PMA. These cells were then pre-treated with either 2.5 μM TPCA-1 or DMSO for 1 h, followed by infection with PRV (MOI of 5) (C) or EMCV (MOI of 10) for 12 hours (D). Western blot analysis was performed to detect mature IL-1β and GSDMD. Sup, supernatant; Lys, lysate. The protein levels were quantified by Image J and normalized to β-actin. The data are representative of results from three independent experiments. Error bars indicate the mean (± SD) of three repeats. *p ≤ 0.05, **p ≤ 0.01, ***p ≤ 0.001; p > 0.05 stands for ns. (TIF) [file ppat.1012915.s004.tif]
